# Assessment of turbulent viscous stress using ICOSA 4D Flow MRI for prediction of hemodynamic blood damage

**DOI:** 10.1038/srep39773

**Published:** 2016-12-22

**Authors:** Hojin Ha, Jonas Lantz, Henrik Haraldsson, Belen Casas, Magnus Ziegler, Matts Karlsson, David Saloner, Petter Dyverfeldt, Tino Ebbers

**Affiliations:** 1 Division of Cardiovascular Medicine, Department of Medical and Health Sciences, Linköping University, Linköping, Sweden; 2 Center for Medical Image Science and Visualization (CMIV), Linköping University, Linköping, Sweden; 3 University of California, San Francisco, San Francisco, California, United States; 4 Division of Applied Thermodynamics and Fluid Mechanics, Department of Management and Engineering (IEI), Linköping University, Linköping, Sweden

## Abstract

Flow-induced blood damage plays an important role in determining the hemodynamic impact of abnormal blood flow, but quantifying of these effects, which are dominated by shear stresses in highly fluctuating turbulent flow, has not been feasible. This study evaluated the novel application of turbulence tensor measurements using simulated 4D Flow MRI data with six-directional velocity encoding for assessing hemodynamic stresses and corresponding blood damage index (BDI) in stenotic turbulent blood flow. The results showed that 4D Flow MRI underestimates the maximum principal shear stress of laminar viscous stress (PLVS), and overestimates the maximum principal shear stress of Reynolds stress (PRSS) with increasing voxel size. PLVS and PRSS were also overestimated by about 1.2 and 4.6 times at medium signal to noise ratio (SNR) = 20. In contrast, the square sum of the turbulent viscous shear stress (TVSS), which is used for blood damage index (BDI) estimation, was not severely affected by SNR and voxel size. The square sum of TVSS and the BDI at SNR >20 were underestimated by less than 1% and 10%, respectively. In conclusion, this study demonstrated the feasibility of 4D Flow MRI based quantification of TVSS and BDI which are closely linked to blood damage.

Abnormal blood flow through stenoses or prosthetic valves has been associated with mechanical damage to blood constituents, leading to hemolysis and thrombosis^1^,^2^,^3^,^4^,^5^. Therefore, quantifying flow-induced blood damage can be useful for assessing the hemodynamic effects caused by obstructed blood flow, and could guide subsequent treatment or surgical interventions for patients.

Flow-induced blood damage is caused by hemodynamic stresses. The effects of laminar viscous shear stresses on blood constituents have been investigated under various scenarios^6^,^7^,^8^. However, blood flow through the aortic valve^9^ or constricted vessels^10^,^11^ can be transitional or turbulent. Crucially, the stresses developed in turbulent flow can be several orders of magnitude larger than the laminar flow^12^, and turbulence-induced blood damage is a major concern for prosthetic devices such as mechanical heart valves^13^.

Estimation of the Reynolds stress, which is a component of the stress tensor derived using the Reynolds-averaged Navier-Stokes (RANS) technique, has been employed when analyzing turbulent effects on blood constituents in flows through prosthetic valves^14^,^15^,^16^. However, the Reynolds stress is a pseudo stress and does not represent a true physical stress^17^. Turbulent viscous shear stress (TVSS), on the other hand, has been derived to directly evaluate the viscous turbulence-related shear stress^18^,^19^. TVSS represents the viscous stress originating from turbulent velocity fluctuations by estimating the spatial gradients of the velocity fluctuations within turbulent eddies, which are on size scales of the same order of magnitude as red blood cells (which are approximately 5–10 μm). The TVSS represents a physical stress linked to hemolysis^18^,^19^.

Previous studies on the relationship between hemodynamics and blood damage were mostly based on numerical simulations^20^,^21^ or *in-vitro* experiments^7^,^8^,^18^,^19^. In particular, previous experiments have analyzed hemolysis under laminar flow within a rotational viscometer, or under turbulent flow created by a stenotic nozzle^7^,^19^. However, neither the rotational viscometer nor the stenotic nozzle flow can successfully recreate the hemodynamic environments for turbulent blood flow in the cardiovascular system. Consequently, the relationship between pathological hemodynamics and hemolysis under turbulent blood flow conditions has not been fully understood^22^,^23^,^24^.

Time-resolved, three-dimensional phase-contrast magnetic resonance imaging, (4D Flow MRI) permits investigations of the hemodynamics of cardiovascular blood flow and provides information needed to study the relationship between pathological hemodynamics and hemolysis under *in-vivo* conditions^25^,^26^. While the conventional 4D Flow MRI technique only measures spatiotemporally averaged velocities and is therefore insensitive to turbulent flow effects, 4D Flow MRI was recently extended to the estimation of the intensity of small-scale velocity fluctuations in turbulent cardiovascular flows^27^,^28^,^29^,^30^. Of late, the 4D Flow MRI turbulence mapping technique was combined with a six-directional icosahedral (ICOSA6) motion-encoding scheme to measure the complete 2^nd^ order symmetrical Reynolds stress tensor^31^,^32^.

This study investigates MRI-based estimation of the effects of turbulent flow on blood constituents using 4D Flow MRI with ICOSA6 motion-encoding. Specifically, we sought to evaluate the assessment of three different hemodynamic stresses: the maximum principal shear stress of laminar viscous stress (PLVS), the maximum principal shear stress of Reynolds stress (PRSS), and turbulent viscous shear stress (TVSS). Additionally, the use of TVSS for blood damage index (BDI) estimation was investigated to confirm the feasibility of stress quantification with Lagrangian integration using 4D Flow MRI. The evaluation was achieved by simulating 4D Flow MRI measurements based on data from computational fluid dynamics (CFD). The effects of spatial resolution and noise on the stress quantification were also analyzed.

## Results

### Quantification of hemodynamic stresses using 4D Flow MRI

**Figure 1:**
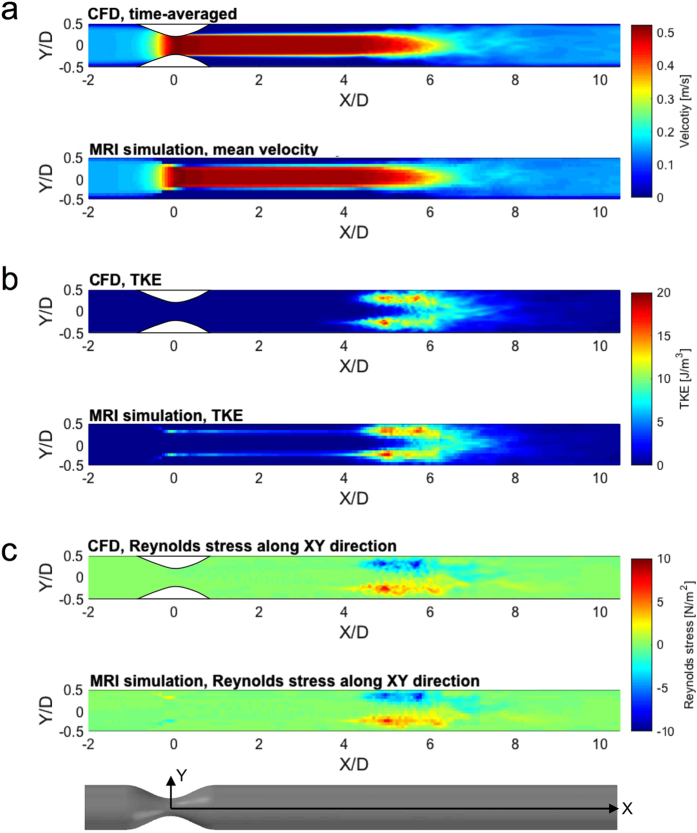
Comparison of CFD and simulated 4D Flow MRI at 75% stenosis with Re = 2000. (**a**) time-averaged velocity field, (**b**) TKE distribution, and (**c**) Reynolds stress component along XY direction. Reynolds stress component along XY direction indicates 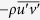 where *u*′ and *v*′ are velocity fluctuation along X and Y axis, respectively. X and Y are normalized by the upstream diameter (D = 14.6 mm). Principal flow direction is toward the positive X direction. The voxel size for simulated MRI was 1 mm. X and Y-axis are depicted in the bottom panel. Z-axis is the through plane direction.

Reference time-averaged velocity fields, turbulent kinetic energy (TKE), and Reynolds stresses were obtained by CFD with large eddy simulation (LES) in a 14.6 mm diameter pipe with cosine-shaped stenoses (60, 75 and 90% reduction in area). A visual comparison of the MRI simulation with 1 mm voxels against the CFD data for the 75% stenosis and Reynolds number (Re) = 2000 case is shown in Fig. 1. Qualitatively, the simulated MRI measurement and the CFD solution agreed well for velocity, TKE, and the primary components of Reynolds stress (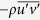; *u*′ and *v*′ are axial and radial directional velocity in the center plane). However, the MRI simulation showed overestimation of TKE and Reynolds stress near the wall and the shear layers of the jet flow, mostly due to partial volume effects. All three shear components of Reynolds stress (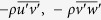, and 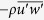) also showed good qualitative agreement with CFD, demonstrating the feasibility of 4D Flow MRI with ICOSA6 encoding for quantification of the full turbulence stress tensor (Supplementary Figure S1)

**Figure 2:**
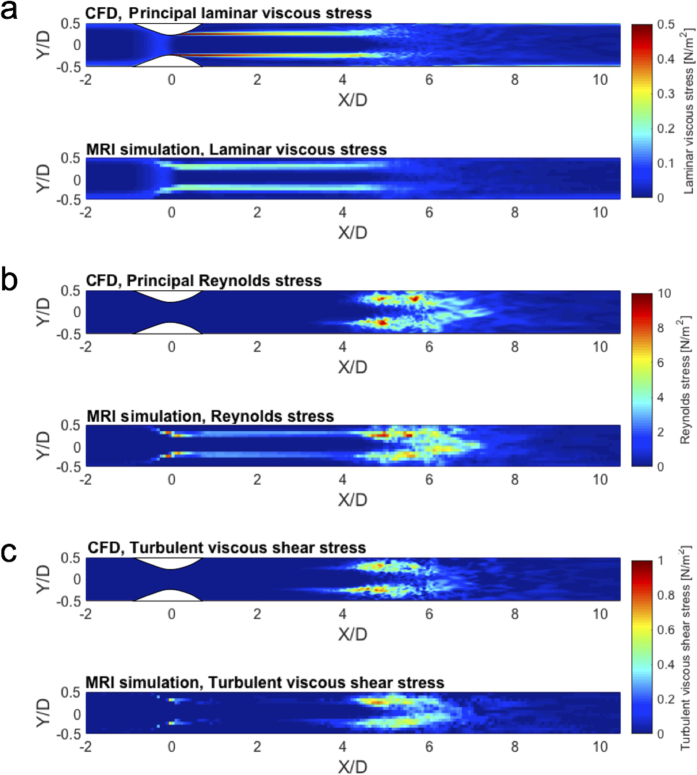
Three different hemodynamic stresses from CFD and simulated 4D Flow MRI at 75% stenosis with Re = 2000. (**a**) maximum principal shear stress of laminar viscous stress (PLVS), (**b**) maximum principal shear stress Reynolds stress (PRSS), and (**c**) turbulent viscous stress (TVSS). Note that laminar viscous stress and Reynolds stress are presented by the maximum principal shear stress. X and Y are normalized by the upstream diameter (D = 14.6 mm). Principal flow direction is toward the positive X direction. The voxel size for simulated MRI was 1 mm.

The MRI simulation showed substantial underestimation of PLVS at 1 mm spatial resolution. The maximum value of the PLVS from MRI simulation was 0.31 N/m^2^, compared to 9.0 N/m^2^ from CFD (Fig. 2a). This is likely related to the spatial resolution, as it is limited when compared to the thickness of the shear layer of the jet flow (Fig. 2a). In contrast, PRSS and TVSS showed a good qualitative agreement (Fig. 2). However, overestimation due to partial volume effects was seen near the wall and at the boundary of the jet flow, where high laminar viscous stress develops.

**Figure 3:**
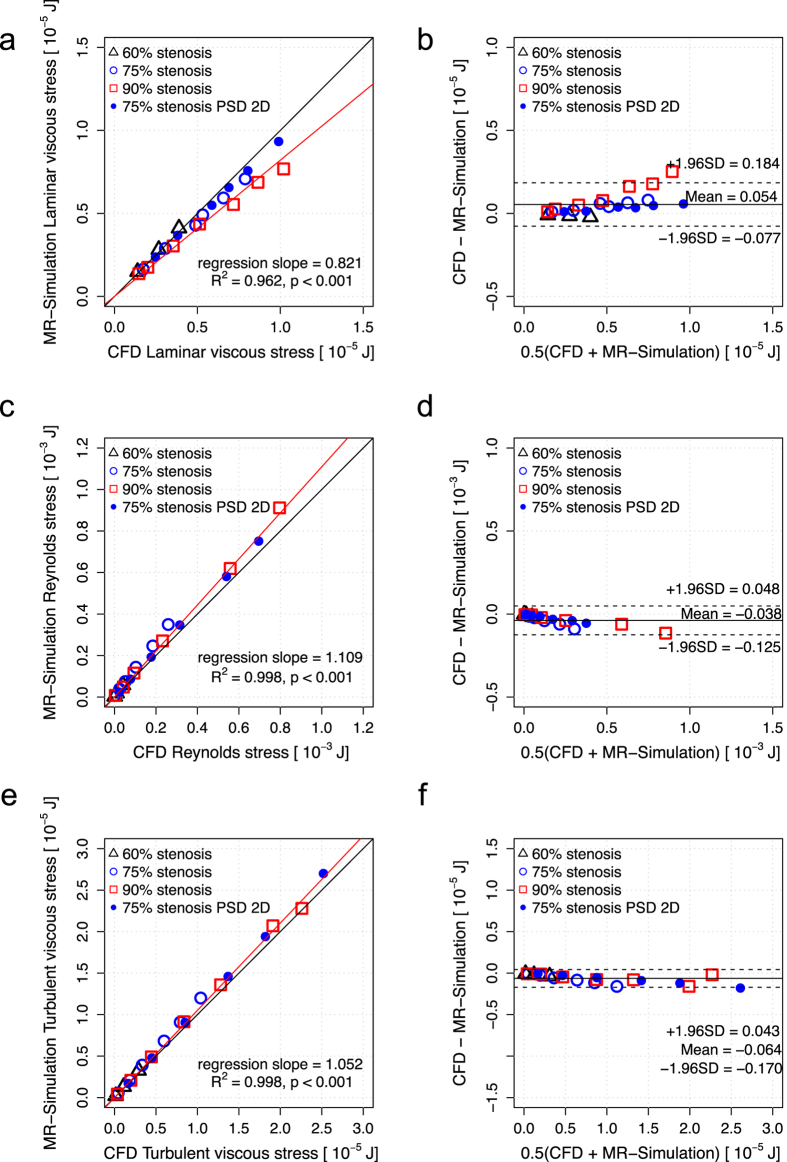
Comparison of PLVS, PRSS and TVSS from CFD and corresponding simulated 4D Flow MRI. (**a**) Correlation of PLVS between CFD and simulated MRI, (**b**) Bland-Altman plot of PLVS. (**c**) Correlation of PRSS between CFD and simulated MRI, (**d**) Bland-Altman plot of PRSS. (**e**) Correlation of TVSS between CFD and simulated MRI, (**f**) Bland-Altman plot of TVSS. Each data point is the volumetric sum of the stress. The voxel size for simulated MRI was 1 mm. Black solid line in (**a**,**c**,**e**) indicates the line of unity. Red solid lines indicate regression lines, which were estimated including all data points with different severity (60–90% reduction in area) and PSD. The solid and dashed lines in (**b**,**d**,**f**) represent mean ± 1.96SD.

Linear regression and Bland-Altman analysis of the volumetric sum of PLVS, PRSS and TVSS in the whole phantom at 1 mm spatial resolution showed that the quantification of hemodynamic stresses using 4D Flow MRI agrees well with ground-truth CFD solutions (Fig. 3). The slope of the regression line for PLVS was 0.821 (p < 0.001, R^2^ = 0.962). The Bland-Altman bias was 0.540 [μJ] with 95% limits of agreement spanning from −0.770 to 1.840 [μJ]. The slope of the regression line for PRSS was 1.109 (p < 0.001) with R^2^ = 0.998. The bias was −0.038 [mJ] with −0.125 to 0.048 [mJ] limits of agreement. The slope of the regression line for TVSS was 1.052 (p < 0.001) with R^2^ = 0.998. The bias was −0.640 [μJ] with −1.700 to 0.430 [μJ] limits of agreement.

**Figure 4:**
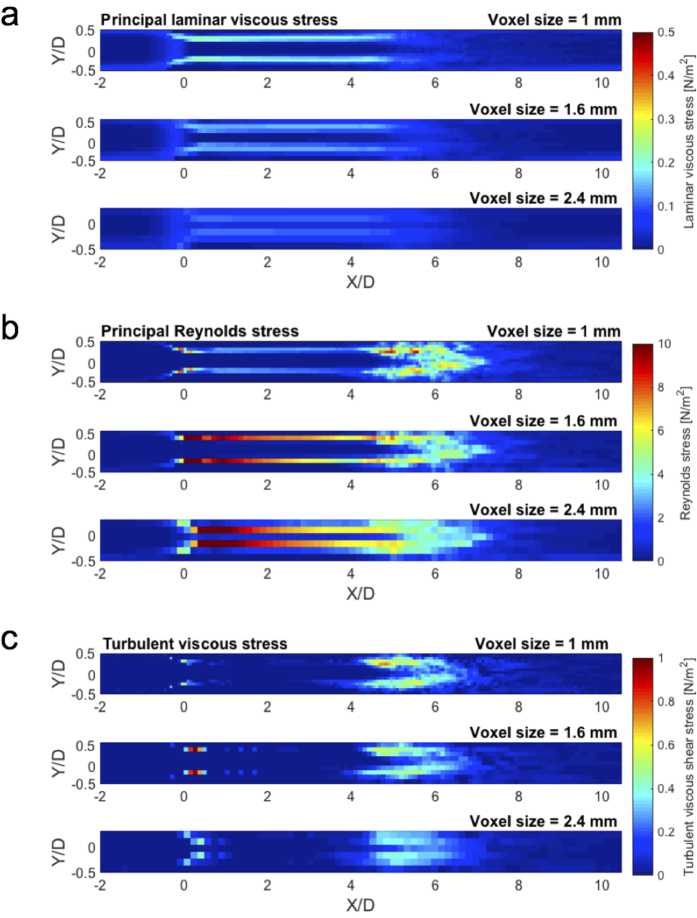
Effect of voxel size on (**a**) PLVS, (**b**) PRSS, and (**c**) TVSS. Results show MRI simulations with voxel sizes of 1 mm, 1.6 mm, and 2.4 mm at 75% stenosis with Re = 2000. Principal flow direction is toward the positive X direction.

**Figure 5:**
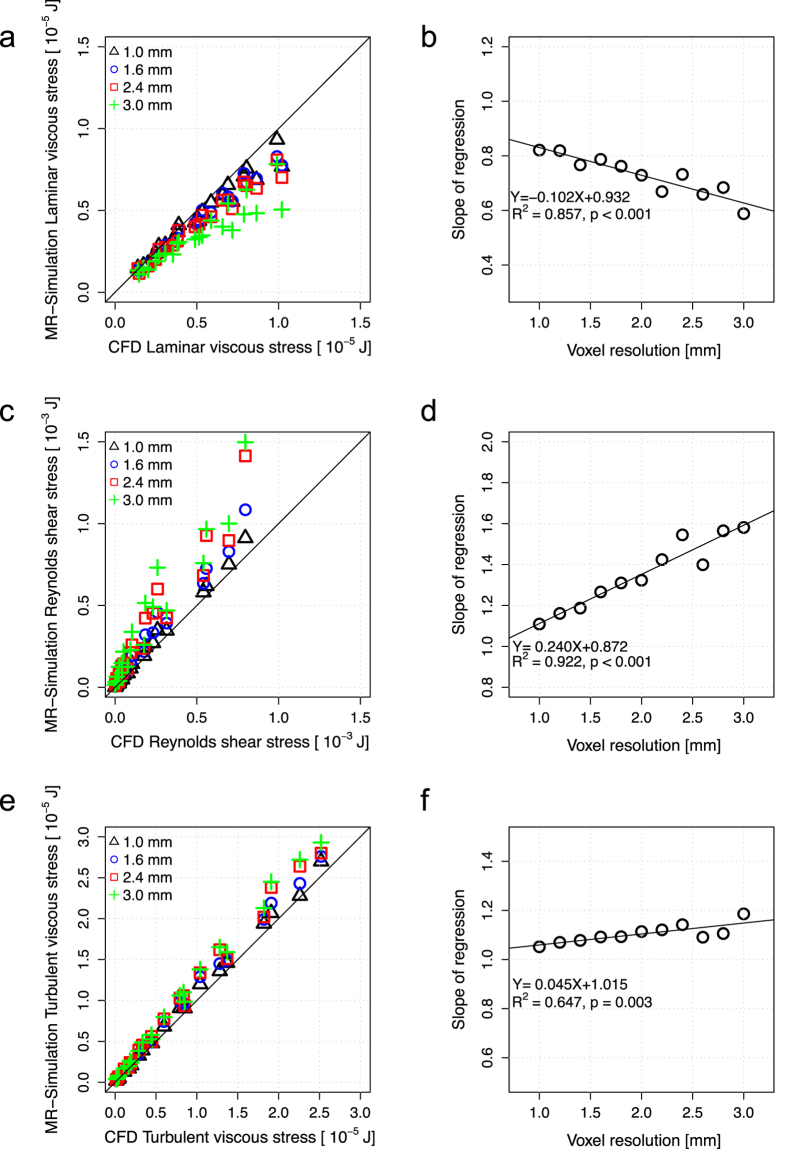
Effect of voxel size on the quantification of PLVS, PRSS, and TVSS. (**a**) Correlation of PLVS between CFD and simulated 4D Flow MRI, (**b**) changes of regression slope with voxel size in relation to PLVS. (**c**) Correlation of PRSS between CFD and simulated MRI, (**d**) changes of regression slope with voxel size in relation to PRSS. (**e**) Correlation of TVSS between CFD and simulated MRI, (**f**) changes of regression slope with voxel size in relation to TVSS. Each data point is the volumetric sum of the stress. Black solid line in (**a**,**c**,**e**) indicates the line of unity. Black solid line in (**b**,**d**,**f**) indicates regression lines in relation between the regression slope and the voxel size. Regression analysis considering all voxel sizes from 1.0–3.0 mm resulted in *τ*_PLVS, MRI_ = 0.729 × *τ*_PLVS, CFD_ + 4.217 × 10^−7^, R^2^ = 0.929, p < 0.001 for PLVS, *τ*_PRSS, MRI_ = 1.352 × *τ*_PRSS, CFD_ + 3.903 × 10^−5^, R^2^ = 0.954, p < 0.001 for PRSS, *τ*_TVSS, MRI_ = 1.104 × *τ*_TVSS, CFD_ + 4.168 × 10^−7^, R^2^ = 0.992, p < 0.001 for TVSS.

The effects of spatial resolution on 4D Flow MRI measurements of PLVS, PRSS, and TVSS are shown in Fig. 4. While the overall magnitude of PLVS decreased as the voxel size was increased, the PRSS at the jet shear layers increased with larger voxel size, resulting in severe overestimation at a voxel size of 2.4 mm. The effect of increased voxel sizes in TVSS was less pronounced. The local maximum value of TVSS increased due to spatial averaging of the stress within the larger voxel. However, no substantial overestimation or underestimation was observed at the boundary of the flow jet. The effects of spatial resolution were consistent for all studied Reynolds numbers (Supplementary Fig. S2). Linear regression analysis showed that the slope of the regression line decreased with larger voxel size for PLVS, increased for PRSS, and increased slightly for TVSS (P < 0.001, Fig. 5). Linear regression and Bland-Altman analyses for each voxel size are summarized in Supplementary Table S1–3.

Linear regression and Bland-Altman analyses, considering all voxel sizes (1–3 mm), showed that the slope of the regression line for PLVS was 0.729 (p < 0.001) with R^2^ = 0.929. The bias was 0.970 [μJ] with limits of agreement: −0.820 and 2.750 [μJ]. Slope of the regression line for PRSS was 1.352 (p < 0.001) with R^2^ = 0.954. The bias was −0.131 [mJ] with limits of agreement: −0.456 and 0.194 [mJ]. Slope of the regression line for TVSS was 1.104 (p < 0.001) with R^2^ = 0.992. The bias was −1.250 [μJ] with limits of agreement: −3.330 and 2.080 [μJ].

**Figure 6:**
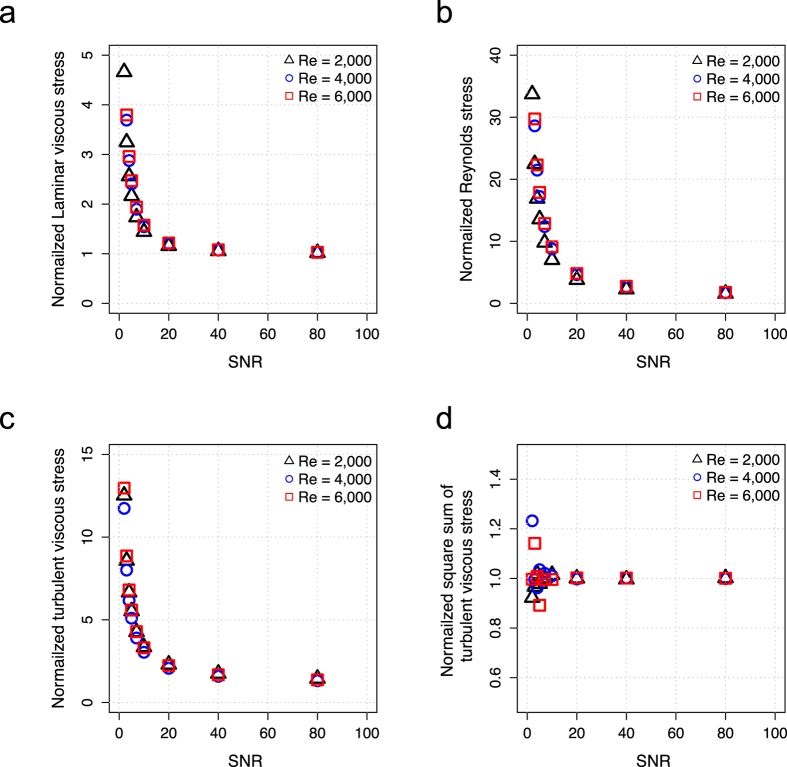
SNR effect on stress quantifications at 75% stenosis with Re = 2000, 4000 and 6000. (**a**) PLVS, (**b**) PRSS, (c) TVSS, and (**c**) square sum of TVSS. Data was normalized to the stress value without added noise (SNR is equal to infinity). Each data point is the mean of ten repetitions at each SNR. SD were not presented for clarity. SD were described in Supplementary Table S4.

Decreased signal-to-noise ratio (SNR) in 4D Flow MRI resulted in approximately zero-mean Gaussian noise in the velocity field, TKE, and Reynolds stress (Supplementary Figure S3–5). However, low SNR was found to introduce a positive bias PLVS, PRSS, and TVSS (Supplementary Fig. S6–8). As a result, the volumetric sums of the stresses gradually increased with decreasing SNR. At SNR = 20, PLVS, PRSS and TVSS were about 1.2, 4.6, and 2.2 times higher than the measurements without noise, respectively (Fig. 6). However, the square sum of TVSS, which is used for BDI estimation, was not severely affected by SNR. The square sum of TVSS at SNR >20 underestimated noise-free results by less than 1% with SD <2% (Fig. 6d). The influence of SNR on the mean and SD of PLVS, PRSS, TVSS, and the square sum of TVSS are summarized in Supplementary Table S4.

### Blood damage estimation using turbulent viscous stress

**Figure 7:**
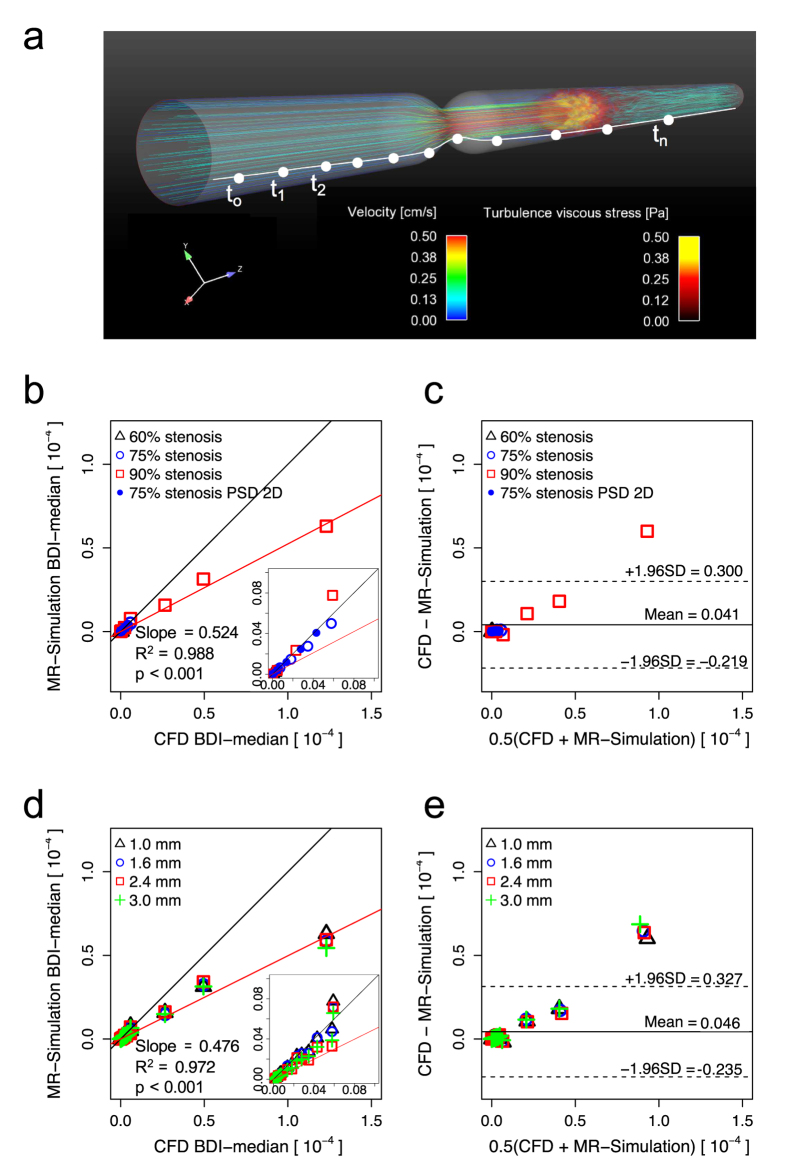
Comparison of BDI estimation from CFD and simulated 4D Flow MRI. (**a**) schematic of BDI estimation based on Lagrangian integration, (**b**) Correlation, and (**c**) Bland-Altman plot of BDI estimation with CFD and simulated MRI for 1 mm voxels. (**d**) Correlation and (**e**) Bland-Altman plot of BDI estimation with CFD and simulated MRI for voxel sizes of 1.0, 1.6, 2.4, and 3.0 mm. White solid line in (**a**) indicates the one representative pathline of the flow. t_0_, t_1_ and t_n_ indicate initial emitting time of the pathline, time at the first step and the n-th step, respectively. Black solid line in (**b**,**d**) indicates the line of unity. Red solid line in (**b**) indicates regression lines, which were estimated including all data points with different stenosis severity and PSD for 1 mm voxels. Red solid line in (**d**) indicates regression lines, which were estimated including all data points with different severity, PSD and voxel sizes (1–3 mm). The solid and dashed lines in (**c**,**e**) represent mean ± 1.96SD.

Blood damage estimation based on Lagrangian integration of turbulent stress along pathlines was analyzed to investigate the feasibility of 4D Flow MRI for conventional BDI estimation (Fig. 7a). Linear regression and Bland-Altman for BDI estimation with TVSS shows that the slope of the regression line at 1 mm spatial resolution was 0.523 (p < 0.001) with R^2^ = 0.988. The bias for 1 mm was 0.041 × 10^−4^ with limits of agreement: −0.219 × 10^−4^ and 0.300 × 10^−4^ (Fig. 7b–c), and the slope of the regression line considering all spatial resolutions (1–3 mm) was 0.476 (p < 0.001) with R^2^ = 0.972. The bias for all spatial resolutions was 0.046 × 10^−4^ with limits of agreement: −0.235 × 10^−4^ and 0.327 × 10^−4^ (Fig. 7d–e). The results showed that 4D Flow MRI estimation for BDI was well correlated to the ground-truth CFD data, though the values are underestimated. Most of the BDI underestimation was observed at 90% stenosis (Fig. 7).

**Figure 8:**
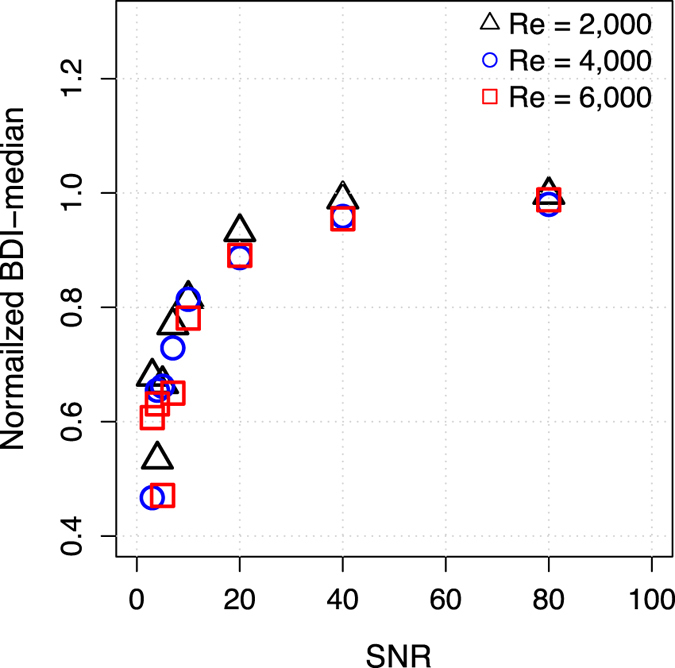
SNR effect on BDI quantifications at 75% stenosis with Re = 2000, 4000, and 6000. Data was normalized to the BDI value without added noise (SNR is equal to infinity). Each data point is the mean of ten repetitions at each SNR. SD were not presented for clarity. SD were described in Supplementary Table S5.

At SNR <10, BDI was underestimated by more than 20% compared to noise-free data (Fig. 8). However, at SNR >20, BDI was underestimated by less than 10% on average with standard deviation <5%. The detailed influence of SNR on BDI quantifications were also summarized in Supplementary Table S5.

## Discussion

A novel application of 4D Flow MRI with ICOSA6 motion-encoding was demonstrated for the assessment of hemodynamic stresses and the corresponding BDI in turbulent blood flow. The major findings are that: (1) PLVS, PRSS and TVSS determined by 4D Flow MRI correlate strongly to ground-truth data within a range of practical spatial resolutions; (2) TVSS is robust against variations in voxel size but PLVS and PRSS were progressively underestimated and overestimated, respectively, as the voxel size increased; (3) a decrease in SNR resulted in the overestimation of the volumetric sum of PLVS, PRSS, and TVSS, but the square sum of TVSS was less affected by noise effects at SNR >20; and, (4) 4D Flow MRI-based estimation of BDI correlated strongly to ground-truth data, although absolute values were underestimated with 4D Flow MRI. When taking the overall findings of the effects of spatial resolution and SNR into account, 4D Flow MRI-based quantification of TVSS and BDI appear to be attractive metrics for the assessment of hemodynamic stresses and associated damage to blood constituents.

Quantifying flow-induced damage to blood constituents can be clinically useful for assessing the hemodynamic impact of obstructed blood flow due to valvular or vascular diseases such as aortic stenosis and aortic coarctation, as well as artificial devices such as artificial heart valves, stents, and grafts. Based on *in-vitro* blood experiments with rotational viscometers, multiple power-law equations and BDIs have been suggested for modeling hemodynamic blood damage and corresponding hemoglobin loss with respect to the magnitude and exposure time of shear stress^7^,^8^,^33^,^34^,^35^. However, it has been difficult to obtain *in-vivo* evidence supporting these models due to the lack of methodology for measuring velocity fields and hemodynamic stresses non-invasively. This study demonstrated that 4D Flow MRI enables the quantification of TVSS and BDI. This methodology could be used for the development and validation of predictive models as well as the quantification of hemodynamic risk generated by obstructed blood flow *in-vivo*.

Our finding that 4D Flow MRI underestimates PLVS is consistent with previous studies^21^,^36^,^37^,^38^. In contrast to the PLVS, PRSS was overestimated with increased voxel sizes. This is because Reynolds stress estimation from 4D Flow MRI assumes that the velocity distribution within the voxel is Gaussian^27^,^28^. Strong velocity gradients within the voxel resulting from local acceleration of the flow or the flow separation can result in intravoxel velocity distributions that differ from a Gaussian distribution^27^,^38^. The overestimation of intravoxel velocity standard deviation (IVSD) and TKE near the vessel wall or jet flow has been observed in previous studies^37^,^39^. It is noteworthy that TVSS was less affected by voxel size than PLVS and PRSS, potentially because the effects of voxel size on the velocity gradients and Reynolds stress cancel out when estimating TVSS.

While the volumetric sum of PLVS, PRSS, and TVSS were substantially overestimated as the SNR decreased, the square sum of TVSS was less influenced by noise. The effects of the SNR on stress quantification were investigated because 4D Flow MRI measurements require trade-offs between scan time and SNR^25^. The calculation of the principal stress and maximum principal shear stress for invariants has non-linear noise transfer given the square sum and square root relationship^40^, although each stress tensor element has Gaussian distributed noise with a zero-mean. Similarly, TVSS was also overestimated at low SNR because it is derived from the square root of the turbulence production term (Fig. 6c). In contrast, the square sum of TVSS was less influenced by the noise because it includes all noise components in the turbulence production, regardless of their signs (Fig. 6d). This is a similar method to that used in TKE estimation for removing the mean bias occurring from the root square estimation^41^.

BDI estimation based on Lagrangian integration of the stress from MRI simulation and CFD was well correlated (R^2^ = 0.988 for 1 mm resolution, R^2^ = 0.977 for all voxel sizes) although the absolute values were underestimated with 4D Flow MRI (Fig. 7). The BDI model was slightly modified to use the square of TVSS to remove the mean bias of TVSS in low SNR. The underestimation was mostly observed in the 90% stenosis cases because turbulent viscous stress for this geometry was locally focused within a thin layer near the stenosis apex (Supplementary Figure S9), compared to the 75% stenosis cases where development occurred within the relatively thick shear layer of the jet flow in the post-stenotic region (Supplementary Figure S10).

Although BDI was strongly underestimated at SNR <10, such poor SNR is not representative of practical measurements. Analysis of the impact of SNR on BDI estimation showed that SNR >20 resulted in the underestimation of BDI by less than 10% with standard deviation below 5% (Fig. 8 and Supplementary Table S5). Considering that conventional 4D Flow MRI acquisitions have SNR >30 without the use of any contrast agent for scan times of 9–23 minutes^42^, the SNR in such practical scenarios will not cause substantial errors on BDI assessments. As our goal was to show the feasibility of stress quantification using Lagrangian integration based on 4D Flow MRI acquisitions, neither the validity of BDI itself, nor comparisons to *in-vitro* studies were within the scope of this study. Further experiments will therefore be necessary to optimize and validate the performance *in-vivo*.

In contrast to the simulated MRI acquisitions, the practical *in-vivo* measurement of 4D Flow MRI can be subject to various types of artifacts. For example, errors in the concomitant field and eddy-current corrections are widely known to degrade the accuracy of the velocity field^43^. Moreover, local signal voids can occur due to prosthetic valves or artificial devices. Therefore, in addition to the effects of spatial resolution and SNR as analyzed in this study, various new sources of errors found in clinical acquisitions can influence the quantification of TVSS and BDI. Although preliminary in nature, the *in-vitro* implementation and demonstration of TVSS quantification using 4D Flow MRI with ICOSA6 encoding showed to be rather straightforward (Supplementary Figure S11 and Supplementary Method). The results obtained in the *in-vitro* test, were in agreement with the simulated acquisitions.

The simulations used in this study are limited by the use of water as a working fluid rather than blood. However, we investigated various regimes of turbulent flow by increasing the Reynolds number of the flow from 500 to 6000. This range includes the majority of turbulent flows found in aortic flow by dynamic similarity^9^. As shown in Supplementary Figure S12, this study analyzed flows in which the Reynolds stress and TVSS approached 500, and 30 N/m^2^, respectively (e.g. 90% stenosis and Re = 6000 case). These stresses are similar in magnitude to the theorized thresholds for red blood cell damage, demonstrating the feasibility of the proposed method in severely turbulent flows.

In contrast to the ideal stenosis model used in this study, some aortic valve stenoses cause flow impingement on the aortic wall. While the quantification of the near-wall turbulence under these circumstances has shown not to be severely affected by the impingement^44^, the velocity gradient near the wall can be underestimated or overestimated due to the limited spatial resolution of 4D Flow MRI^21^. Consequently, the assessment of TVSS and BDI, which are principally combinations of the Reynolds stress and the velocity gradient, can be less accurate for these stenoses.

Extending this methodology for pulsatile flows is of great interest, given the pulsatile nature of cardiovascular flow. The quantification of TVSS is fundamentally similar to the quantification of TKE. Since both definitions of TKE and TVSS do not contain any terms related to temporal velocity variation, their quantifications do not require further modifications regardless of flow pulsatility. Previously, TKE measurements for a pulsatile blood flow were compared with CFD, and the result was well agreed without any additional errors from the pulsatile nature of the blood flow^11^. Following the demonstration in the pulsatile blood flow, the TKE measurement has been successfully used for *in-vivo* patients, providing the temporal variations of TKE during the cardiac cycle^45^. Therefore, although the methodology in this study was demonstrated only for the steady flow, extending TVSS quantification for a pulsatile blood flow will be straightforward and will provide spatio-temporal variation of TVSS, localizing the hemodynamic risk of the blood damage.

In conclusion, this study evaluated the assessment of three different hemodynamic stresses using 4D Flow MRI with ICOSA6 motion-encoding. The estimation of TVSS from the Reynolds stress tensor was robust to variations in voxel size and noise, compared to the PLVSS and PRSS. The assessment of BDI was demonstrated using Lagrangian integration of the TVSS, showing that 4D Flow MRI with ICOSA6 encoding can assess turbulent stresses and corresponding BDI, which are closely linked to the blood damage.

## Methods

### Computational fluid dynamics

The geometry and flow conditions of the stenosis flow for the numerical simulation were previously described in Casas *et al*.^37^. The stenotic model was designed by following a cosine-form formula^46^,^47^. In this study, three different stenosis severities (60, 75, and 90% reduction in area), and two different poststenotic dilatations (PSD) with one and two times of the upstream diameter (D = 14.6 mm) were investigated. The stenosis geometries and flow conditions for the present study are summarized in Supplementary Table S6.

Non-pulsatile steady flow was simulated numerically by solving the Navier-Stokes equations in ANSYS CFX 14.5. High quality anisotropic hexahedral cells for the computational mesh were constructed using ANSYS ICEM 14.5. The number of cells was on the order of 10–18 million, depending on Reynolds number (Re)^37^. The non-dimensional wall distance y+ was less than unity to ensure high resolution near the wall, sufficient to resolve the near wall turbulent flow^10^,^11^,^48^,^49^.

Flow was computed using large eddy simulation (LES), a technique that has been validated with both laser Doppler velocimetry and direct numerical simulations^49^. The simulation employed the Wall-Adapting Local Eddy-viscosity (WALE) subgrid scale model^10^, and the numerical schemes were second order accurate. The time step for resolving turbulent flow fluctuations was 50 μs for Re <4000, and 25 μs for Re = 5000 and 6000, which have been shown to be sufficient for stenotic flow in similar flow regimes^10^,^11^,^48^,^49^. Data sampling started after one second of flow time to avoid initial transient effects.

A fully developed Poiseuille flow and a constant static pressure were given as inlet and outlet boundary conditions, respectively. The inlet and outlet were placed at four and twenty-one times the upstream diameter (D = 14.6 mm) upstream and downstream of the stenosis, respectively. A rigid wall obeying the no-slip condition was used for the simulation. The working fluid was water with a constant density of 997 kg/m^3^ and dynamic viscosity of 8.899 × 10^−4^ kg/m·s.

### 4D Flow MRI simulation

Simulated 4D Flow MRI measurements allow for comprehensive studies on the effects of multiple MRI parameters. In this study, simulations were performed using a previously reported method that was extended to include the ICOSA6 sequence^37^. Isotropic voxel sizes of 1 to 3 mm were simulated at 0.2 mm intervals based on the velocity data from CFD. The velocity within the voxel was estimated using Gaussian weighting with a coefficient w_j_ based on the distance of velocities from numerical simulation with respect to the center of the voxel^37^,^44^,^50^:


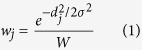


where *d* is the distance of the *j*-th data point to the center of the voxel, *W* is the total weight within the voxel (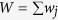), and *σ* is the the variance of the Gaussian function, which is set based on the spatial resolution ∆*z* as ∆*z*/2.35^37^.

To simulate the time averaged velocity field in 4D Flow MRI, the velocity within each voxel in all directions was calculated by averaging of all velocity data from the transient LES solution using a Gaussian weighting function. More than 400 unique velocity vectors from the LES solution were averaged for each voxel. Using this method for simulation, the spatial regularization and temporal averaging effects seen with 4D Flow MRI can be mimicked while limiting other artifacts, such as those seen during practical measurements (e.g. background phase errors or signal voids).

The velocity distributions *s(v)* of the velocity *v* within the voxel were obtained by estimating the probability density function of the velocities within the voxel. A total of 20–30 LES solutions at 10–20 ms intervals were used to estimate the intravoxel velocity distribution. The MRI signal *S(k*_*v*_) for velocity distribution *s(v)* was calculated by the Fourier-transformation as follows^27^,^28^:


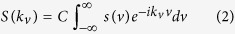


where C is a constant scaling factor influenced by the relaxation parameter, spin density, receiver gain, etc. *k*_*v*_ is the amount of flow sensitivity, which is related to the velocity encoding parameter (VENC) as *k*_*v*_ = *π/VENC*. The IVSD along the *i*-direction *σ*_*i*_ was estimated by the magnitude ratio between the reference signal without velocity encoding *S(0)* and the signal with the velocity encoding along the *i*-direction *S*_*i*_*(k*_*v*_) as follows^27^,^28^:


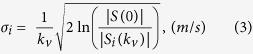


To investigate the effect of SNR on the quantification of hemodynamic stress, SNRs between 2–80 were simulated by adding Gaussian noise on the velocity field and signal magnitude in Eq. [2], where SNR was estimated as the 3*σ* of the Gaussian noise distribution per original velocity or signal magnitude. Therefore, the effect of SNR on both the mean velocity and IVSD were investigated.

This study simulated 4D Flow MRI with the ICOSA6 sequence, which employs six directional velocity encodings. Velocity and *σ*_*i*_ for each velocity encoding were simulated following the velocity encoding directions shown in Supplementary Table S7. Least square solutions for the six velocity encodings and IVSD were calculated to extract three velocity components (u, v, and w), three IVSDs (*σ*_*x*_, *σ*_*y*_ and *σ*_*z*_), and three off-diagonal Reynolds stress tensor components, based on the relationship between the ICOSA6 velocity encoding and the flow components in Cartesian coordinates (Supplementary Table S8)^51^.

### Total kinetic energy (TKE)

For each voxel of the stenosis flow, TKE was calculated from the intravoxel velocity standard deviation in each direction as follows^17^:


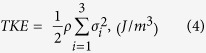


### Quantification of hemodynamic stresses

Laminar viscous stress assumes that the flow is laminar. In laminar flow, the viscous stress tensor is defined as^19^:


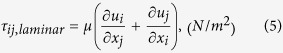


where *μ* is the dynamic viscosity, u_i_ and u_j_ are the velocity along i-th and j-th directions (i, j) = [x, y, or z]).

In turbulent flow, Reynolds stress is often used to represent the turbulent stress as:


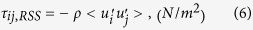


where *ρ* is the fluid density, *u*′ is the velocity fluctuation, and bracket <·> indicates ensemble average. In the present study, maximum principal shear stress was used to represent the laminar (PLVS) and Reynolds stresses (PRSS) as invariants as^6^:


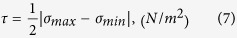


where *σ*_*max*_ and *σ*_*min*_ are maximum and minimum principal stress of the stress tensor.

As an alternative to Reynolds stress, which is a statistical representation and not a physical shear stress, turbulent viscous stress (TVSS), which represents the viscous stress exerted by turbulent velocity fluctuations, can be estimated as follows^18^,^19^:


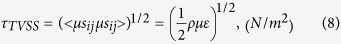


where s_ij_ is the strain rate tensor of velocity fluctuations, *ε* is the turbulence dissipation rate of the flow. The direct calculation *ε* is not trivial because the strain rate tensor of the velocity fluctuations cannot be obtained using 4D Flow MRI. Alternatively, the dynamic balance between turbulence dissipation and turbulent production can be employed as^52^:


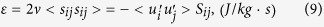


where S_ij_ is the strain rate tensor of mean velocity field. Therefore, TVSS can be estimated from the product of the Reynolds stress and strain rate tensor of the mean velocity field, which are both possible to measure with 4D Flow MRI.

TVSS can be overestimated at low SNR because it has been derived from the square root of the turbulence production term *ε*. Locally negative values in the turbulence production term due to random noise can cause the turbulence production to be undefined in TVSS. Eliminating all undefined elements in the TVSS, the net influence of the noise will cause the overestimation of TVSS. Therefore, in this study, the square sum of TVSS was employed to reduce the influence on the parameter from noise by including all noise components in the turbulence production, regardless of their signs.

### Shear-induced blood damage index (BDI)

BDI estimation in the present study is based on the previous work by Grigioni *et al*.^35^, which employed Lagrangian integration of stress along flow pathlines based on a modified power-law model. In this study, a minor modification was applied to minimize the influence of measurement noise on BDI estimation.

Conventional blood damage index (BDI) is empirically expressed as:


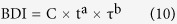


where t is the exposure time, and τ is the fluid shear stress. The most popular coefficients for BDI have been used (a = 0.785, b = 2.416, and C = 3.62 × 10^−5^)^35^. By defining a *mechanical dose D* function as:


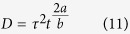



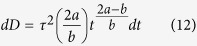


Assuming τ is constant during dt. D(t) can be expressed as discrete summation of stress along the pathline:


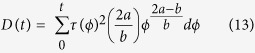


Assuming *D* is zero at the initial condition. Then, accumulation of BDI at each integration step (ΔBDI) can be also expressed by discrete summation of *D*:


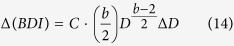



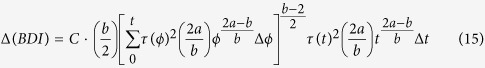


therefore, the total BDI can be obtained by integrating BDI along the pathline (see also Grigioni *et al*.^35^ for the detailed derivation of BDI).

In this study, turbulent viscous stress was used for the BDI estimation. To calculate the pathline integration, 2000 pathlines were uniformly emitted from the inlet of the stenosis channel using Ensight (CEI, Apex, NC). Lagrangian integration of the stress and median values of total BDI distribution at the outlet of the stenosis were analyzed in Matlab (Mathworks, Natick, Mass).

### Statistical Analysis

In this study, volumetric integration of stress was analyzed as one of the stress indices. Linear regression was analyzed to assess the relationship between the ground-truth CFD solution and 4D Flow MRI simulation. The slope and coefficient of determination of the regression (R^2^) were calculated using RStudio (RStudio, Inc., Boston, MA). Bland-Altman analysis was also used to evaluate the agreement between the LES solution and MRI simulation.

## Additional Information

**How to cite this article**: Ha, H. *et al*. Assessment of turbulent viscous stress using ICOSA 4D Flow MRI for prediction of hemodynamic blood damage. *Sci. Rep.* **6**, 39773; doi: 10.1038/srep39773 (2016).

**Publisher's note:** Springer Nature remains neutral with regard to jurisdictional claims in published maps and institutional affiliations.

## Supplementary Material

Supplementary Information
