# Supplementary material for: Assessment of turbulent viscous stress using ICOSA 4D Flow MRI for prediction of hemodynamic blood damage
Source: Sci Rep. 2016 Dec 22;6:39773. doi: 10.1038/srep39773 (PMC5177919; doi:10.1038/srep39773)
Supplement: Supplementary Information [file srep39773-s1.pdf]

Assessment of turbulent viscous stress using ICOSA 4D Flow MRI for prediction of hemodynamic blood damage

Hojin Ha[1,2], Jonas Lantz[1,2], Henrik Haraldsson[3], Belen Casas[1,2], Magnus Ziegler[1,2], Matts Karlsson[2,4], David Saloner[3], Petter Dyverfeldt[1,2], Tino Ebbers[1,2]

[1]Division of Cardiovascular Medicine, Department of Medical and Health Sciences, Linköping University, Linköping, Sweden.
[2]Center for Medical Image Science and Visualization (CMIV), Linköping University, Linköping, Sweden.
[3] University of California, San Francisco, San Francisco, California, United States.
[4]Division of Applied Thermodynamics and Fluid Mechanics, Department of Management and Engineering (IEI), Linköping University, Linköping, Sweden.

Supplementary information

Corresponding Author: Hojin Ha

Phone: +46-762693607

E-mail: hojin.ha@liu.se

Running title: Assessment of turbulent viscous stress using 4D flow MRI.

Number of words: 4025 words

Number of figures: 8 figures

Supplementary Tables

Table S1. Effect of voxel resolution on maximum principal shear stress of laminar viscous stress (PLVS) estimation

Voxel resolution [mm]	$\tau_{PLVS,\ MRI} = a \times \tau_{PLVS,\ CFD} + b$				$\tau_{PLVS,\ CFD} - \tau_{PLVS,\ MRI}$
	a	b	R^2	p-value	(Mean±1.96SD, 10^{-5} J)
1.0	0.821	3.816×10^{-7}	0.962	<0.001	0.054±0.140
1.2	0.819	3.604×10^{-7}	0.083	<0.001	0.056±0.114
1.4	0.767	4.007×10^{-7}	0.980	<0.001	0.079±0.139
1.6	0.787	3.763×10^{-7}	0.980	<0.001	0.072±0.130
1.8	0.762	3.943×10^{-7}	0.976	<0.001	0.082±0.143
2.0	0.729	4.275×10^{-7}	0.975	<0.001	0.096±0.159
2.2	0.669	4.621×10^{-7}	0.938	<0.001	0.123±0.201
2.4	0.732	4.104×10^{-7}	0.969	<0.001	0.096±0.161
2.6	0.659	4.851×10^{-7}	0.938	<0.001	0.126±0.205
2.8	0.684	4.681×10^{-7}	0.957	<0.001	0.115±0.187
3.0	0.588	4.721×10^{-7}	0.866	<0.001	0.164±0.255

Table S2. Effect of voxel resolution on maximum principal shear stress of Reynolds shear stress (PRSS) estimation

Voxel resolution [mm]	$\tau_{\text{PRSS, MRI}} = a \times \tau_{\text{PRSS, CFD}} + b$				$\tau_{\text{PRSS, CFD}} - \tau_{\text{PRSS, MRI}}$ (Mean±1.96SD, 10^{-3} J)
	a	b	R^2	p-value	
1.0	1.109	9.786×10^{-6}	0.998	<0.001	-0.038±0.087
1.2	1.161	1.531×10^{-5}	0.996	<0.001	-0.057±0.129
1.4	1.187	1.466×10^{-5}	0.994	<0.001	-0.064±0.151
1.6	1.266	2.393×10^{-5}	0.992	<0.001	-0.093±0.208
1.8	1.310	3.448×10^{-5}	0.988	<0.001	-0.116±0.248
2.0	1.323	3.363×10^{-5}	0.987	<0.001	-0.118±0.258
2.2	1.424	4.700×10^{-5}	0.985	<0.001	-0.158±0.331
2.4	1.545	3.511×10^{-5}	0.975	<0.001	-0.178±0.431
2.6	1.399	7.422×10^{-5}	0.978	<0.001	-0.179±0.326
2.8	1.565	7.195×10^{-5}	0.969	<0.001	-0.220±0.455
3.0	1.581	6.890×10^{-5}	0.969	<0.001	-0.221±0.466

Table S3. Effect of voxel resolution on turbulent viscous shear stress (TVSS) estimation

Voxel resolution [mm]	$\tau_{\text{TVSS, MRI}} = a \times \tau_{\text{TVSS, CFD}} + b$				$\tau_{\text{TVSS, CFD}} - \tau_{\text{TVSS, MRI}}$ (Mean±1.96SD, 10^{-5} J)
	a	b	R^2	p-value	
1.0	1.052	2.224×10^{-7}	0.998	<0.001	-0.064±0.107
1.2	1.070	2.661×10^{-7}	0.998	<0.001	-0.082±0.130
1.4	1.078	2.886×10^{-7}	0.997	<0.001	-0.091±0.144
1.6	1.092	3.420×10^{-7}	0.997	<0.001	-0.108±0.165
1.8	1.093	4.074×10^{-7}	0.996	<0.001	-0.115±0.175
2.0	1.114	3.613×10^{-7}	0.997	<0.001	-0.127±0.192
2.2	1.121	4.767×10^{-7}	0.996	<0.001	-0.144±0.212
2.4	1.142	4.384×10^{-7}	0.993	<0.001	-0.157±0.257
2.6	1.091	6.034×10^{-7}	0.992	<0.001	-0.133±0.197
2.8	1.106	7.192×10^{-7}	0.990	<0.001	-0.157±0.227
3.0	1.186	4.576×10^{-7}	0.995	<0.001	-0.194±0.305

Table S4. Effect of SNR on fluid-dynamic stress quantification

Reynolds number	SNR	PLVS		PRSS		TVSS		Square sum of TVSS	
		mean	SD	mean	SD	mean	SD	mean	SD
2000	Inf	1.000	0.000	1.000	0.000	1.000	0.000	1.000	0.000
2000	80	1.018	0.000	1.564	0.001	1.448	0.003	1.002	0.004
2000	40	1.054	0.000	2.279	0.002	1.750	0.005	0.997	0.006
2000	20	1.159	0.000	3.807	0.003	2.302	0.012	1.000	0.020
2000	10	1.444	0.002	7.006	0.011	3.353	0.012	1.015	0.046
2000	7	1.737	0.003	9.806	0.016	4.266	0.021	1.001	0.078
2000	5	2.166	0.004	13.573	0.027	5.526	0.023	0.979	0.125
2000	4	2.563	0.003	16.895	0.019	6.664	0.024	1.018	0.134
2000	3	3.247	0.004	22.478	0.031	8.584	0.037	0.967	0.286
2000	2	4.663	0.006	33.723	0.039	12.521	0.067	0.923	0.642
3000	Inf	1.000	0.000	1.000	0.000	1.000	0.000	1.000	0.000
3000	80	1.012	0.000	1.733	0.001	1.468	0.004	1.001	0.003
3000	40	1.043	0.000	2.644	0.002	1.776	0.006	1.003	0.007
3000	20	1.138	0.001	4.572	0.007	2.307	0.011	0.998	0.018
3000	10	1.414	0.002	8.574	0.007	3.339	0.015	0.999	0.035
3000	7	1.704	0.002	12.060	0.012	4.265	0.023	1.001	0.052
3000	5	2.133	0.002	16.739	0.019	5.541	0.022	1.022	0.100
3000	4	2.531	0.003	20.859	0.023	6.677	0.044	0.978	0.203
3000	3	3.219	0.006	27.793	0.041	8.646	0.044	1.101	0.264
3000	2	4.642	0.005	41.649	0.068	12.591	0.066	0.918	0.547
4000	Inf	1.000	0.000	1.000	0.000	1.000	0.000	1.000	0.000
4000	80	1.025	0.000	1.709	0.002	1.313	0.003	0.999	0.005
4000	40	1.072	0.000	2.636	0.003	1.581	0.005	1.002	0.004
4000	20	1.202	0.001	4.629	0.006	2.067	0.009	0.998	0.014
4000	10	1.545	0.001	8.767	0.008	3.037	0.017	1.011	0.040
4000	7	1.897	0.003	12.374	0.012	3.899	0.024	1.020	0.061
4000	5	2.407	0.003	17.220	0.024	5.098	0.017	1.035	0.101
4000	4	2.878	0.004	21.473	0.027	6.164	0.025	0.962	0.160
4000	3	3.692	0.005	28.604	0.028	8.005	0.034	0.995	0.257
4000	2	5.358	0.007	42.929	0.037	11.737	0.046	1.232	0.468
5000	Inf	1.000	0.000	1.000	0.000	1.000	0.000	1.000	0.000
5000	80	1.027	0.000	1.725	0.002	1.357	0.004	1.000	0.003
5000	40	1.078	0.000	2.652	0.003	1.657	0.004	0.998	0.005
5000	20	1.214	0.001	4.635	0.008	2.207	0.005	1.002	0.010
5000	10	1.573	0.001	8.740	0.015	3.277	0.014	0.983	0.025
5000	7	1.938	0.002	12.327	0.011	4.237	0.009	1.007	0.033
5000	5	2.467	0.003	17.136	0.021	5.559	0.020	1.034	0.074
5000	4	2.956	0.004	21.355	0.012	6.732	0.029	0.991	0.127
5000	3	3.792	0.005	28.417	0.039	8.744	0.023	1.051	0.156
5000	2	5.514	0.008	42.688	0.041	12.792	0.072	0.789	0.663
6000	Inf	1.000	0.000	1.000	0.000	1.000	0.000	1.000	0.000
6000	80	1.027	0.000	1.763	0.001	1.370	0.004	1.001	0.003
6000	40	1.079	0.000	2.736	0.003	1.676	0.003	1.002	0.006
6000	20	1.215	0.001	4.809	0.003	2.228	0.013	1.002	0.020
6000	10	1.575	0.002	9.114	0.009	3.311	0.016	0.996	0.028
6000	7	1.940	0.003	12.865	0.013	4.283	0.022	0.995	0.058
6000	5	2.471	0.004	17.884	0.021	5.591	0.021	0.892	0.086
6000	4	2.958	0.004	22.303	0.034	6.804	0.033	1.008	0.134
6000	3	3.795	0.005	29.730	0.046	8.861	0.046	1.142	0.278
6000	2	5.516	0.006	44.599	0.053	12.964	0.055	0.997	0.439

Table S5. Effect of SNR on BDI quantification

Severity	Reynolds number	SNR	Normalized BDI	Standard deviation
75	2000	Inf	1.000	0.000
75	2000	80	0.997	0.017
75	2000	40	0.989	0.013
75	2000	20	0.932	0.033
75	2000	10	0.815	0.062
75	2000	7	0.769	0.135
75	2000	5	0.666	0.148
75	2000	4	0.534	0.614
75	2000	3	0.679	0.714
75	2000	2	-0.234	2.239
75	3000	Inf	1.000	0.000
75	3000	80	0.993	0.009
75	3000	40	0.971	0.021
75	3000	20	0.921	0.040
75	3000	10	0.820	0.035
75	3000	7	0.750	0.101
75	3000	5	0.705	0.128
75	3000	4	0.492	0.257
75	3000	3	0.853	0.564
75	3000	2	0.544	1.555
75	4000	Inf	1.000	0.000
75	4000	80	0.980	0.010
75	4000	40	0.959	0.023
75	4000	20	0.887	0.027
75	4000	10	0.814	0.066
75	4000	7	0.729	0.135
75	4000	5	0.662	0.123
75	4000	4	0.655	0.509
75	4000	3	0.467	0.795
75	4000	2	1.789	1.657
75	5000	Inf	1.000	0.000
75	5000	80	0.983	0.011
75	5000	40	0.954	0.019
75	5000	20	0.910	0.044
75	5000	10	0.763	0.057
75	5000	7	0.712	0.097
75	5000	5	0.715	0.125
75	5000	4	0.412	0.624
75	5000	3	0.741	0.865
75	5000	2	1.778	2.100
75	6000	Inf	1.000	0.000
75	6000	80	0.988	0.013
75	6000	40	0.955	0.030
75	6000	20	0.891	0.047
75	6000	10	0.781	0.063
75	6000	7	0.650	0.091
75	6000	5	0.471	0.309
75	6000	4	0.631	0.429
75	6000	3	0.607	0.896
75	6000	2	0.249	1.709

Table S6. Geometry and flow conditions for the numerical simulation and 4D Flow MRI simulation

Severity[a] (%)	PSD[b]	Reynolds number
60	-	1000
60	-	2000
60	-	3000
75	-	1000
75	-	2000
75	-	3000
75	-	4000
75	-	5000
75	-	6000
90	-	500
90	-	1000
90	-	2000
90	-	3000
90	-	4000
90	-	5000
90	-	6000
75	2D	1000
75	2D	2000
75	2D	3000
75	2D	4000
75	2D	5000
75	2D	6000

[a]Severity is the percentage of area reduction at the stenosis apex.
[b]Post-stenotic dilatation (PSD), defined as a ratio between a diameter at the post-stenosis and upstream diameter.

Table S7. Flow encoding scheme with ICOSA6 sequence

Number of encoding	Conventional 4D flow MRI	ICOSA encoding
0	0	0
1	$\Delta M_1(x)$	$\Delta M_1(\cos\theta^{b}\cdot x + \sin\theta\cdot y)$
2	$\Delta M_1(y)$	$\Delta M_1(\cos\theta\cdot x - \sin\theta\cdot y)$
3	$\Delta M_1(z)$	$\Delta M_1(\cos\theta\cdot y + \sin\theta\cdot z)$
4	NA[a]	$\Delta M_1(\cos\theta\cdot y - \sin\theta\cdot z)$
5	NA	$\Delta M_1(\sin\theta\cdot x + \cos\theta\cdot z)$
6	NA	$\Delta M_1(\sin\theta\cdot x - \cos\theta\cdot z)$

[a]NA; not-applicable, [b] θ for the present study is about 31.17º, which corresponds to $\cos\theta$ = 0.8507 and $\sin\theta$ = 0.5257

Table S8. Velocity and intravoxel turbulence parameters from ICOSA6 sequence

Number of encoding	Velocity component	Intravoxel standard deviation
1	$V_1 = \cos\theta \cdot u + \sin\theta \cdot v$	$\sigma_1^2 = \cos^2\theta \cdot (\sigma_x^2) + \sin^2\theta \cdot (\sigma_y^2) + 2 \cdot (\cos\theta) \cdot (\sin\theta) \cdot (\langle u'v' \rangle)$
2	$V_2 = \cos\theta \cdot u - \sin\theta \cdot v$	$\sigma_2^2 = \cos^2\theta \cdot (\sigma_x^2) + \sin^2\theta \cdot (\sigma_y^2) - 2 \cdot (\cos\theta) \cdot (\sin\theta) \cdot (\langle u'v' \rangle)$
3	$V_3 = \cos\theta \cdot v + \sin\theta \cdot w$	$\sigma_3^2 = \cos^2\theta \cdot (\sigma_y^2) + \sin^2\theta \cdot (\sigma_z^2) + 2 \cdot (\cos\theta) \cdot (\sin\theta) \cdot (\langle v'w' \rangle)$
4	$V_4 = \cos\theta \cdot v - \sin\theta \cdot w$	$\sigma_4^2 = \cos^2\theta \cdot (\sigma_y^2) + \sin^2\theta \cdot (\sigma_z^2) - 2 \cdot (\cos\theta) \cdot (\sin\theta) \cdot (\langle v'w' \rangle)$
5	$V_5 = \sin\theta \cdot u + \cos\theta \cdot w$	$\sigma_5^2 = \sin^2\theta \cdot (\sigma_x^2) + \cos^2\theta \cdot (\sigma_z^2) + 2 \cdot (\cos\theta) \cdot (\sin\theta) \cdot (\langle u'w' \rangle)$
6	$V_6 = \sin\theta \cdot u - \cos\theta \cdot w$	$\sigma_6^2 = \sin^2\theta \cdot (\sigma_x^2) - \cos^2\theta \cdot (\sigma_z^2) + 2 \cdot (\cos\theta) \cdot (\sin\theta) \cdot (\langle u'w' \rangle)$

[*] u,v and w indicate the velocity component in three orthogonal direcitons along x,y, and z.

[**] <u’v’>, <v’w’> and <u’w’> indicate the Reynolds stress component

Supplementary Figures

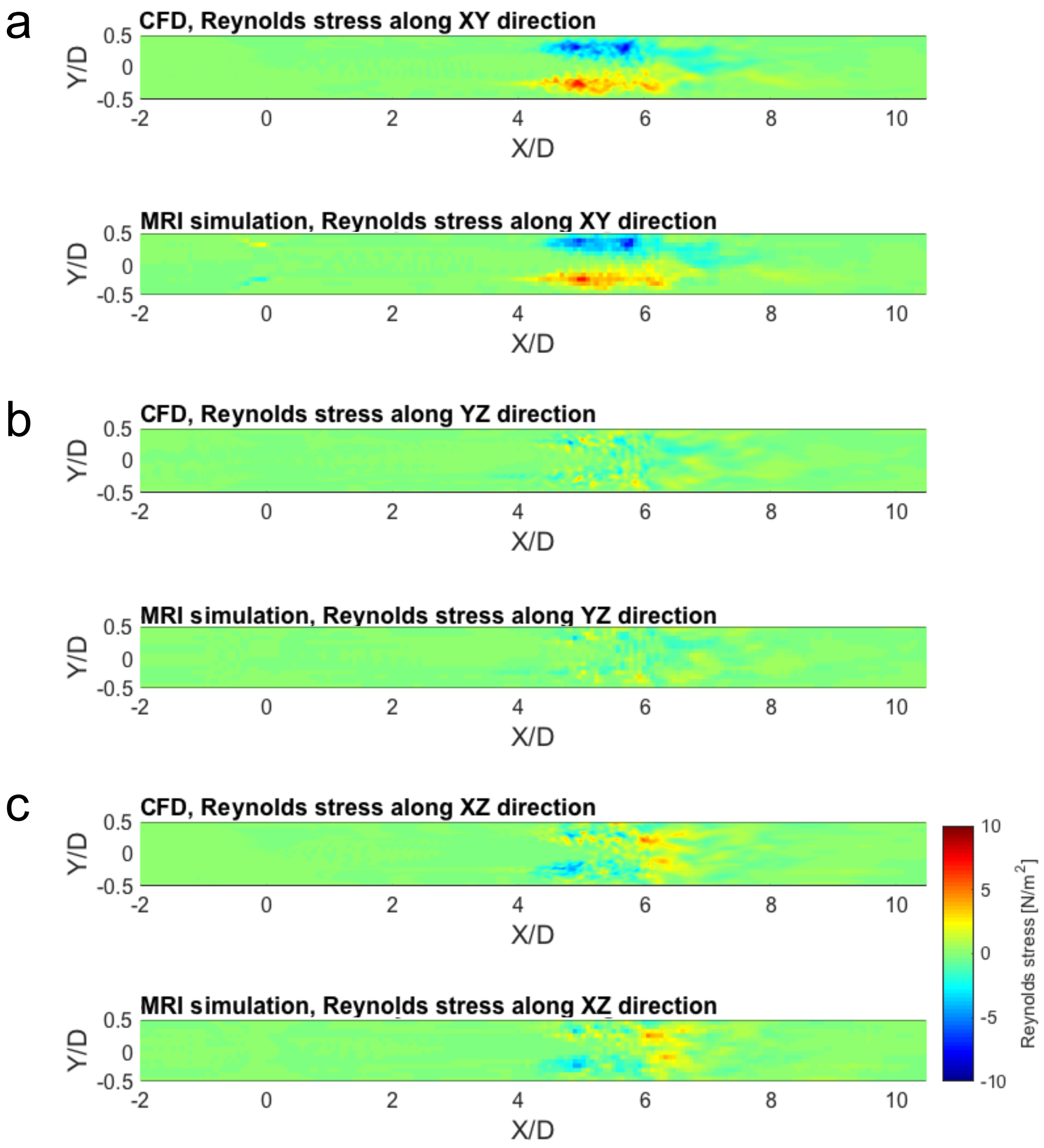

Figure S1. Comparison of Reynolds stress with CFD at the XY measurement plane (75% stenosis with Re = 2000). (a) Reynolds stress component along XY direction (-$\rho\overline{u'v'}$), (a) Reynolds stress component along YZ direction (-$\rho\overline{v'w'}$) and (c) (a) Reynolds stress component along XZ direction (-$\rho\overline{u'w'}$), where u', v' and w' are velocity fluctuation along X, Y and Z axis, respectively. X and Y are normalized by the upstream diameter (D = 14.6 mm). Principal flow direction is toward the positive X direction. The voxel size for MRI simulation was set to 1 mm. Note that the figure only shows the XY directional components because those are identical with the XZ directional components due to the symmetricity of stenosis channel.

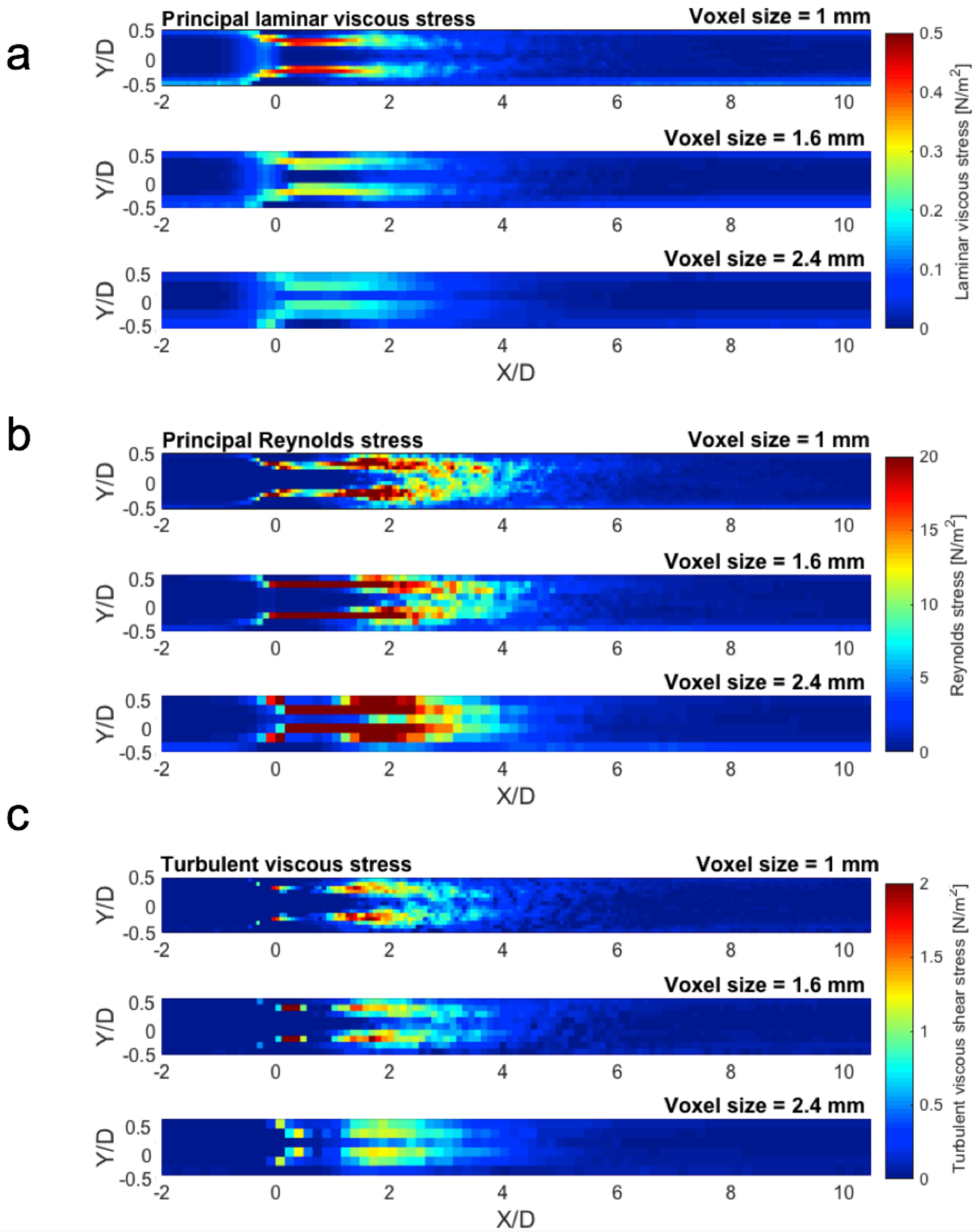

Figure S2. Effect of voxel resolution on (a) PLVS, (b) PRSS and (c) TVSS. Results shows MRI simulation with the voxel size of 1 mm, 1.6 mm and 2.4 mm at 75% stenosis with Re = 4000. Principal flow direction is toward the positive X direction.

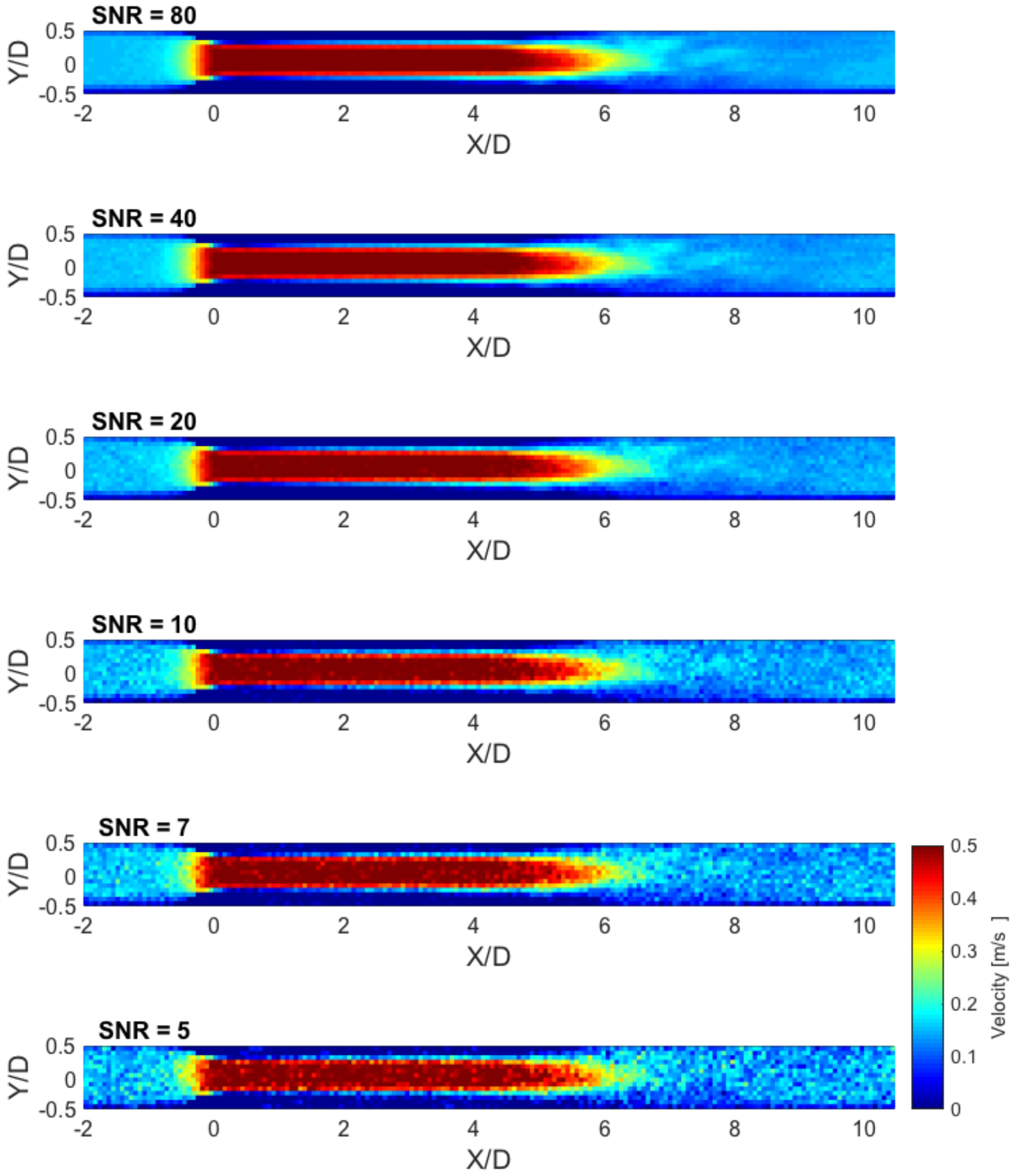

Figure S3. Effect of SNR on the time-averaged velocity field at 75% stenosis with Re = 2000. X and Y are normalized by the upstream diameter (D = 14.6 mm). Principal flow direction is toward the positive X direction. The voxel size for MRI simulation was set to 1 mm.

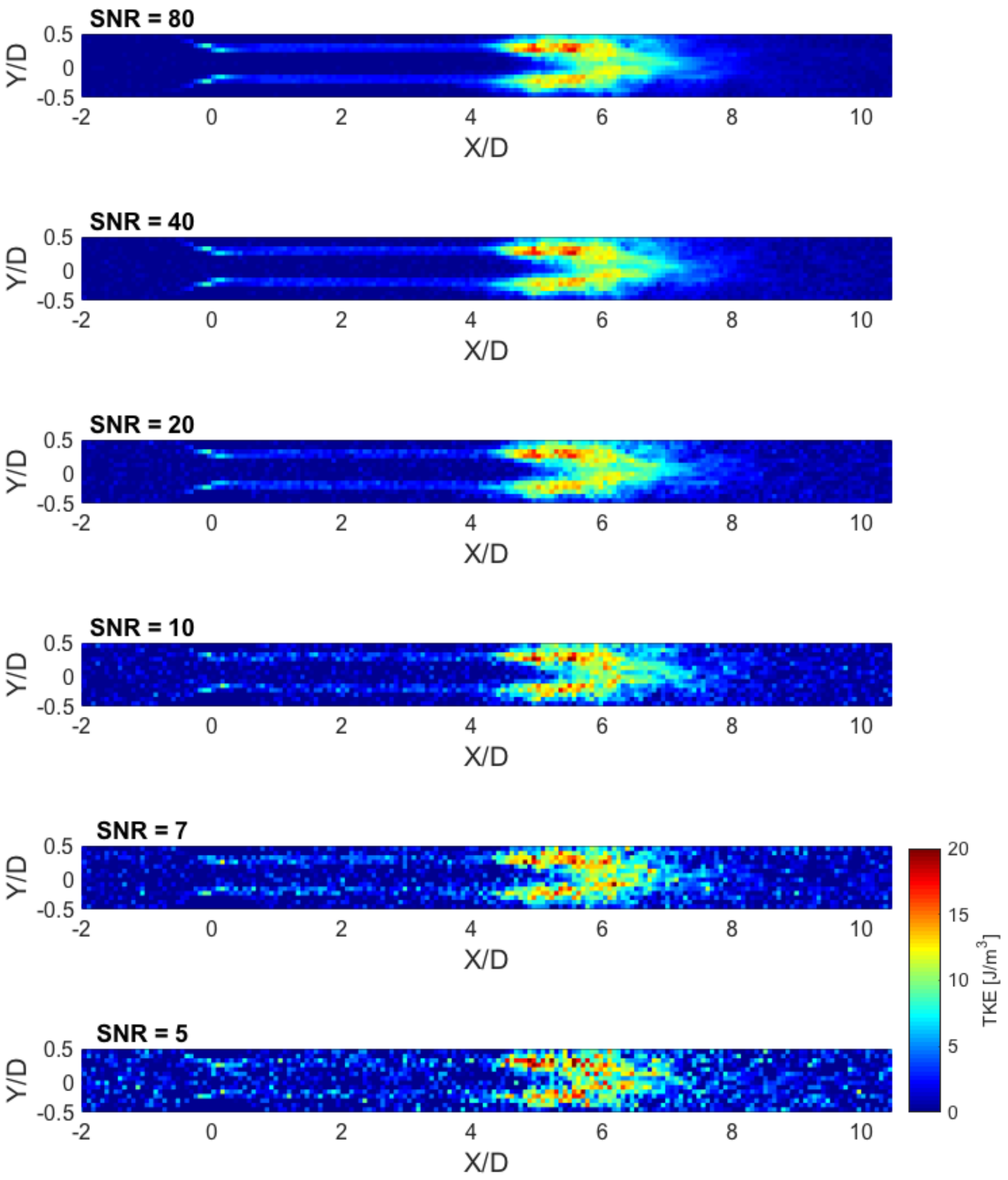

Figure S4. Effect of SNR on TKE field at 75% stenosis with Re = 2000. X and Y are normalized by the upstream diameter (D = 14.6 mm). Principal flow direction is toward the positive X direction. The voxel size for MRI simulation was set to 1 mm.

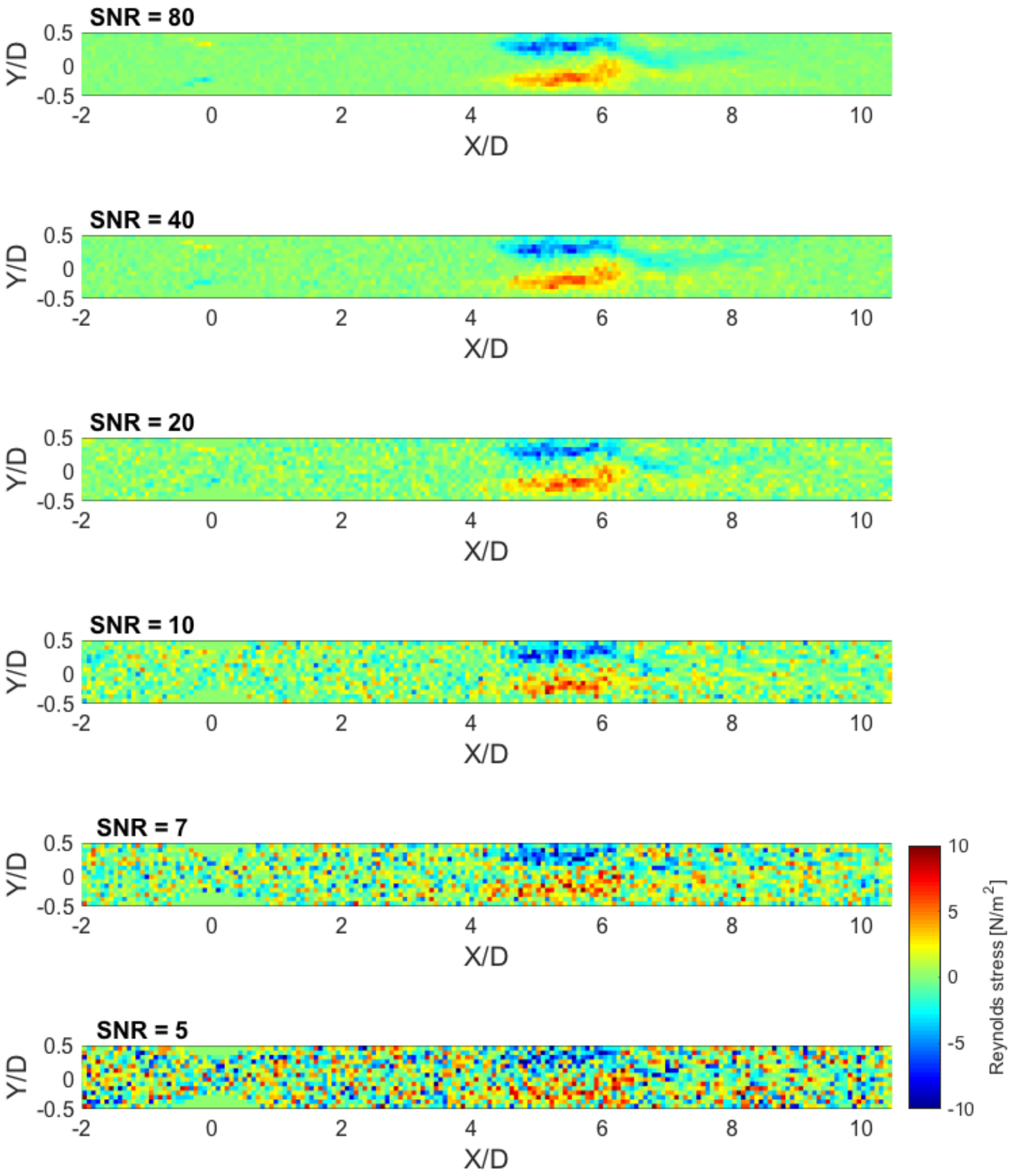

Figure S5. Effect of SNR on Reynolds stress along XY direction at 75% stenosis with Re = 2000. Reynolds stress component along XY direction indicates -$\rho\overline{u'v'}$ where u' and v' are velocity fluctuation along X and Y axis, respectively. X and Y are normalized by the upstream diameter (D = 14.6 mm). Principal flow direction is toward the positive X direction. The voxel size for MRI simulation was set to 1 mm.

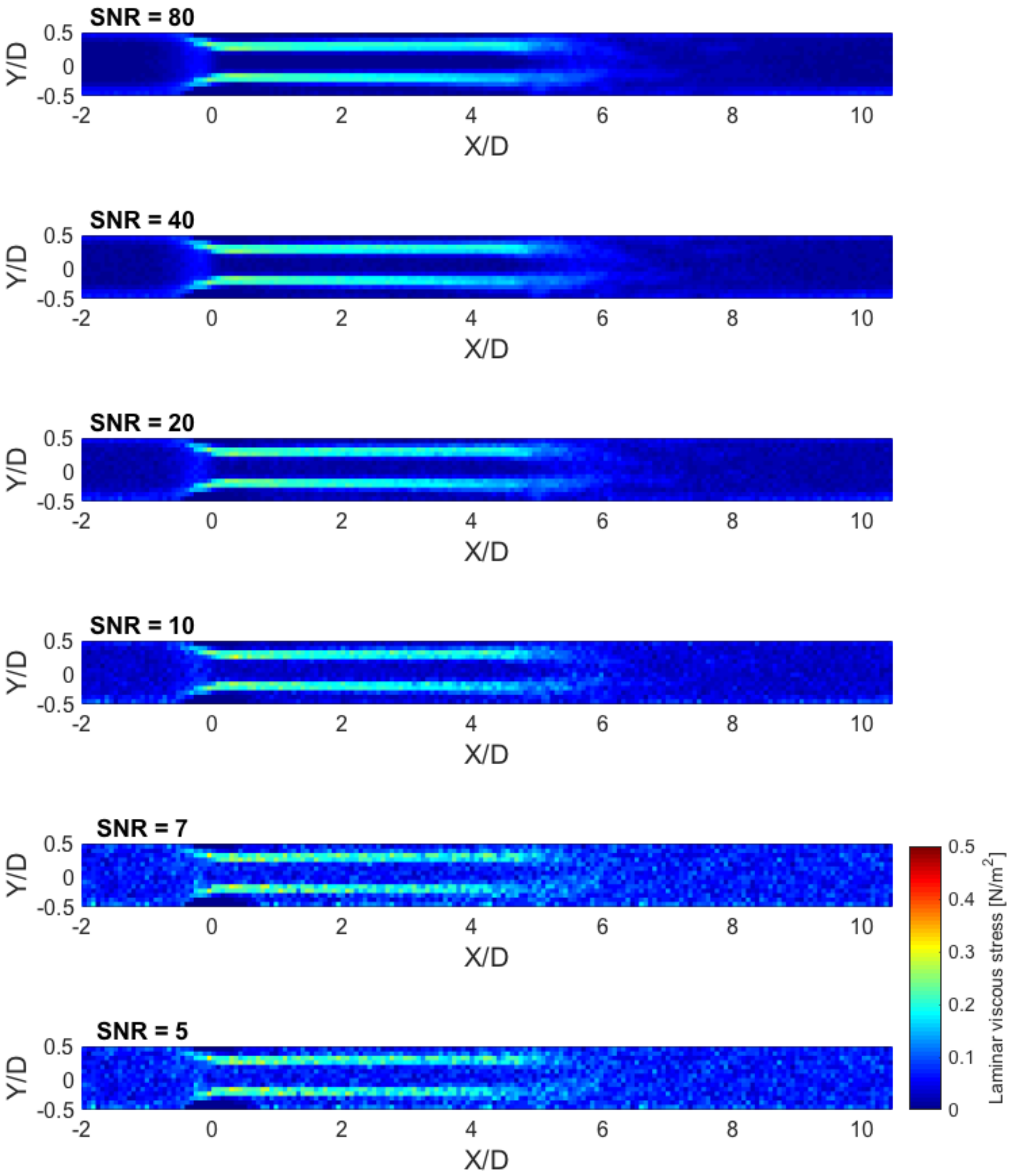

Figure S6. Effect of SNR on PLVS at 75% stenosis with Re = 2000. X and Y are normalized by the upstream diameter (D = 14.6 mm). Principal flow direction is toward the positive X direction. The voxel size for MRI simulation was set to 1 mm.

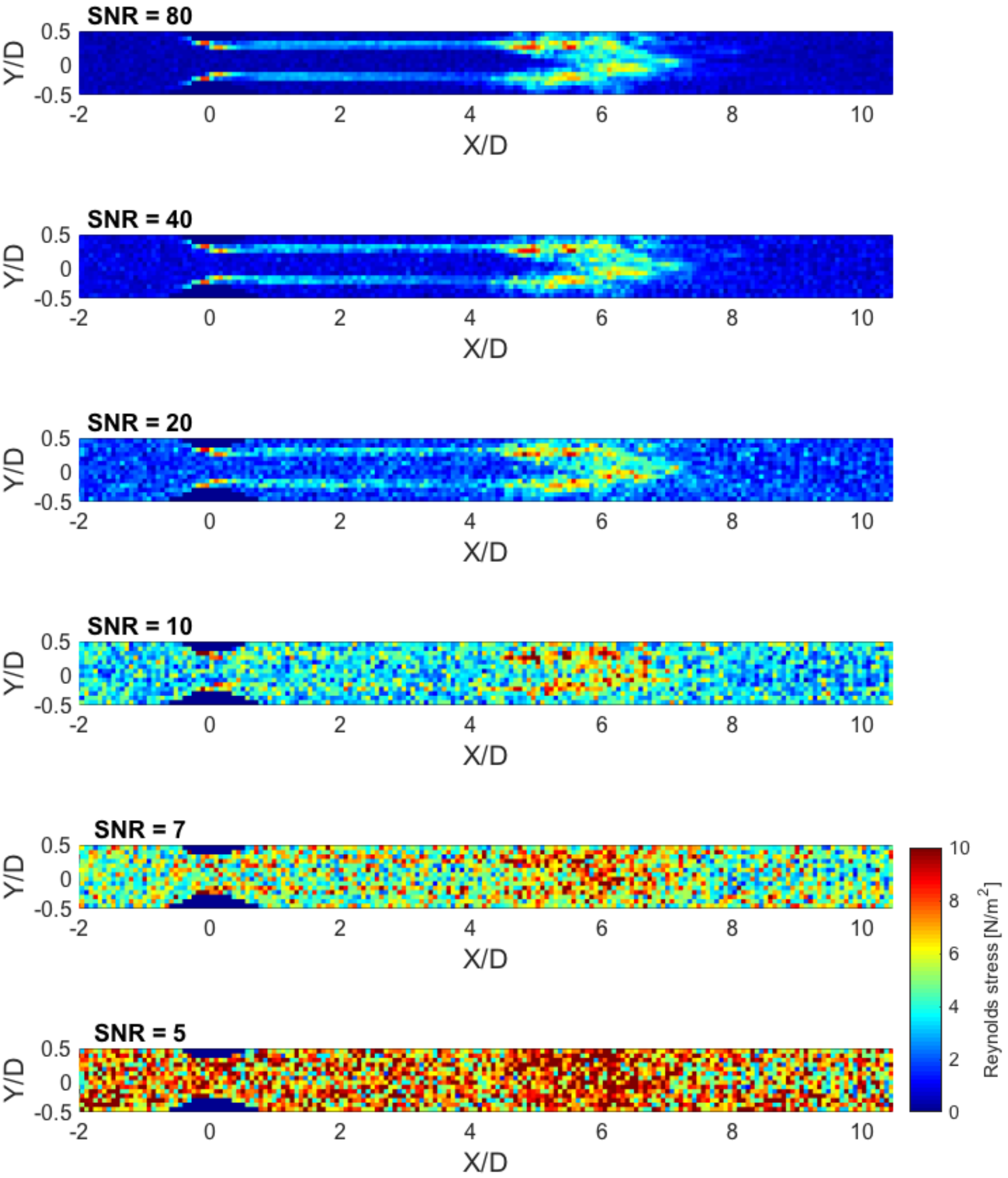

Figure S7. Effect of SNR on PRSS at 75% stenosis with Re = 2000. X and Y are normalized by the upstream diameter (D = 14.6 mm). Principal flow direction is toward the positive X direction. The voxel size for MRI simulation was set to 1 mm.

.

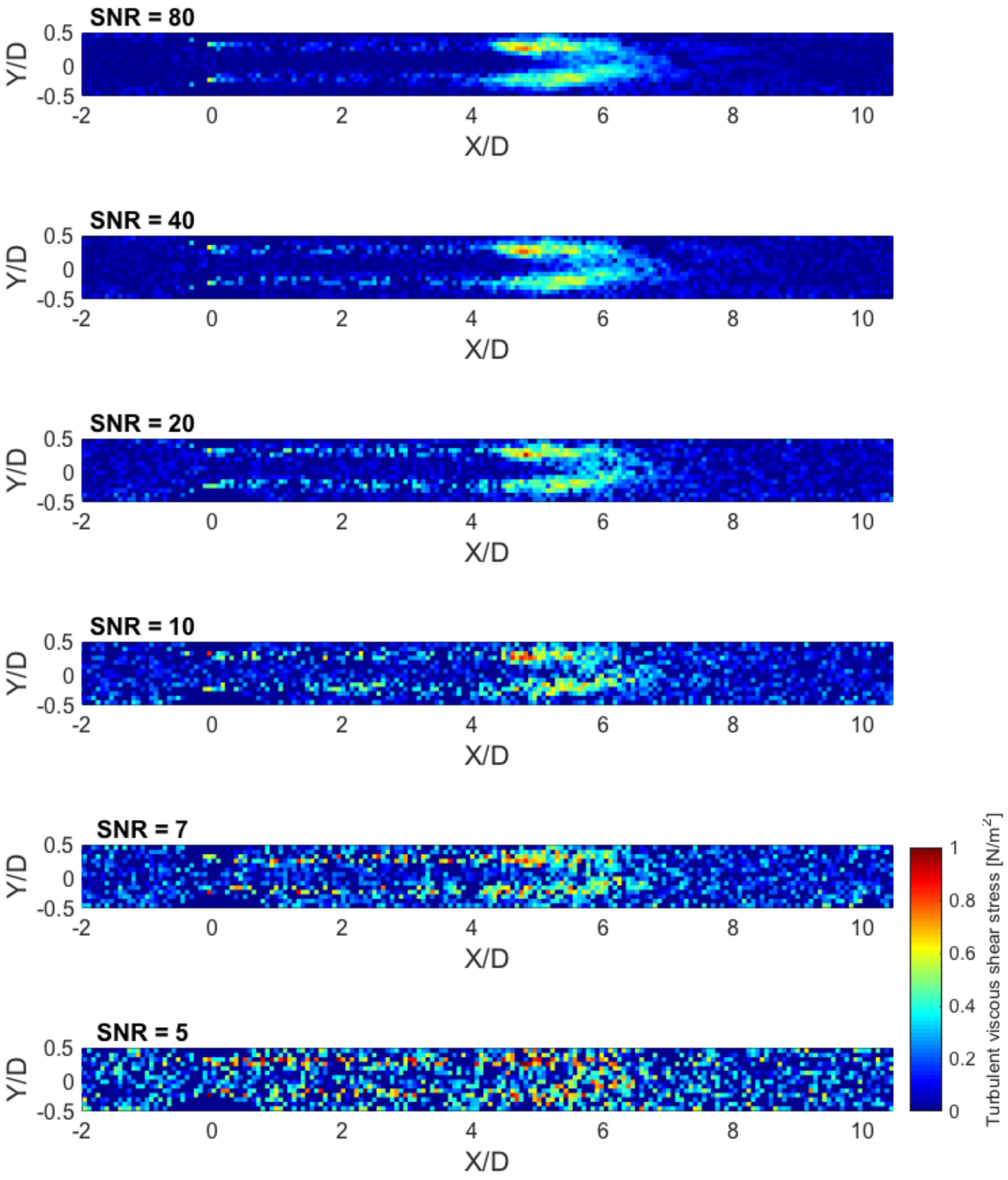

Figure S8. Effect of SNR on TVSS at 75% stenosis with Re = 2000. X and Y are normalized by the upstream diameter (D = 14.6 mm). Principal flow direction is toward the positive X direction. The voxel size for MRI simulation was set to 1 mm.

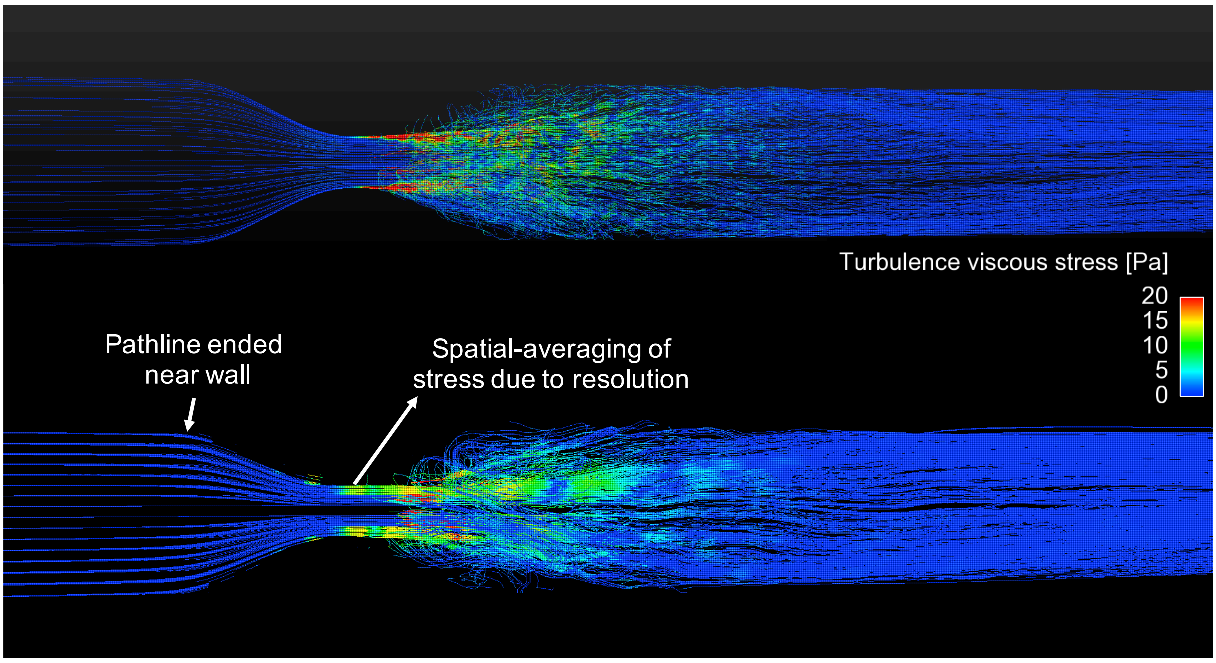

Figure S9. Pathlines flow visualization at 90% stenosis with Re = 6000 obtained from CFD (upper panel) and 4D Flow MRI simulation (lower panel). Pathlines were colored by TVSS. Note that turbulent viscous stress in 90% stenosis with Re = 6000 is mostly focused on thin layer near the stenosis apex. In contrary to the volumetric integration of stress, BDI estimation with MRI at 90% with Re = 6000 could be underestimated because the spatial-averaging of stress values due to the limited resolution influences the stress-history in BDI estimation.

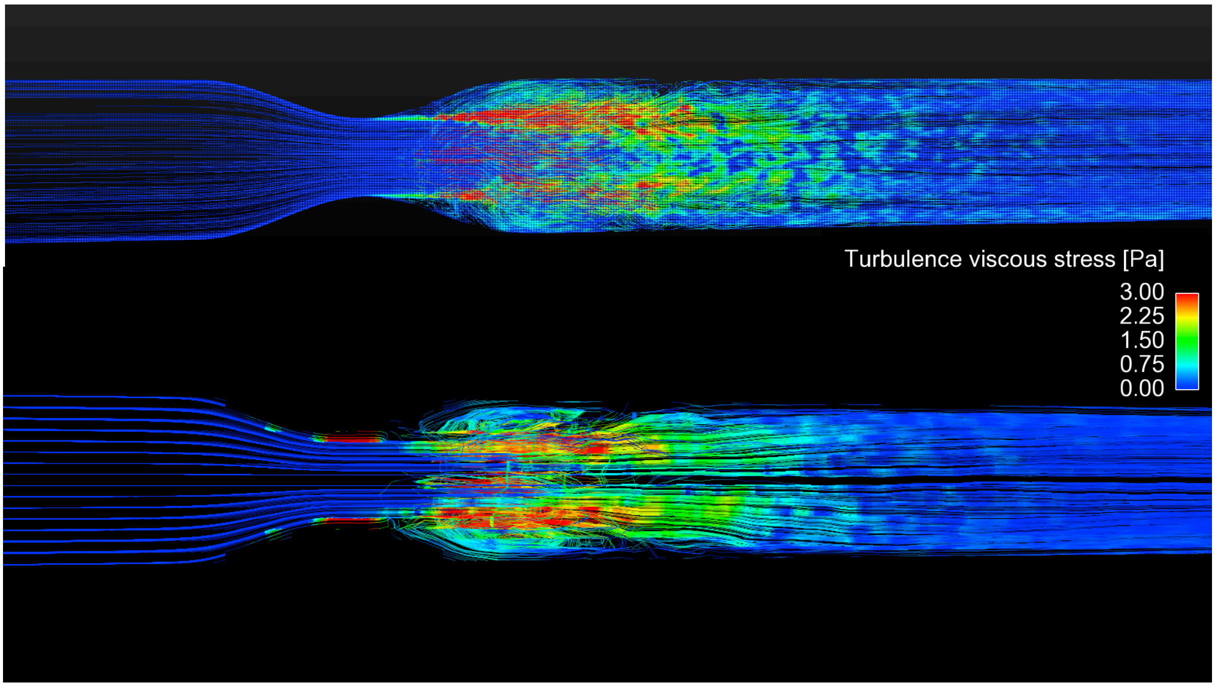

Figure S10. Pathlines flow visualization at 75% stenosis with Re = 6000 obtained from CFD (upper panel) and 4D Flow MRI simulation (lower panel). Pathlines were colored by TVSS. In contrary to 90% stenosis, BDI estimation with MRI for 75% stenosis can be relatively free from the underestimation because turbulent viscous stress is distributed within relatively wider layer at the post-stenosis. High turbulent viscous stress at the stenosis apex was caused by the partial volume effect.

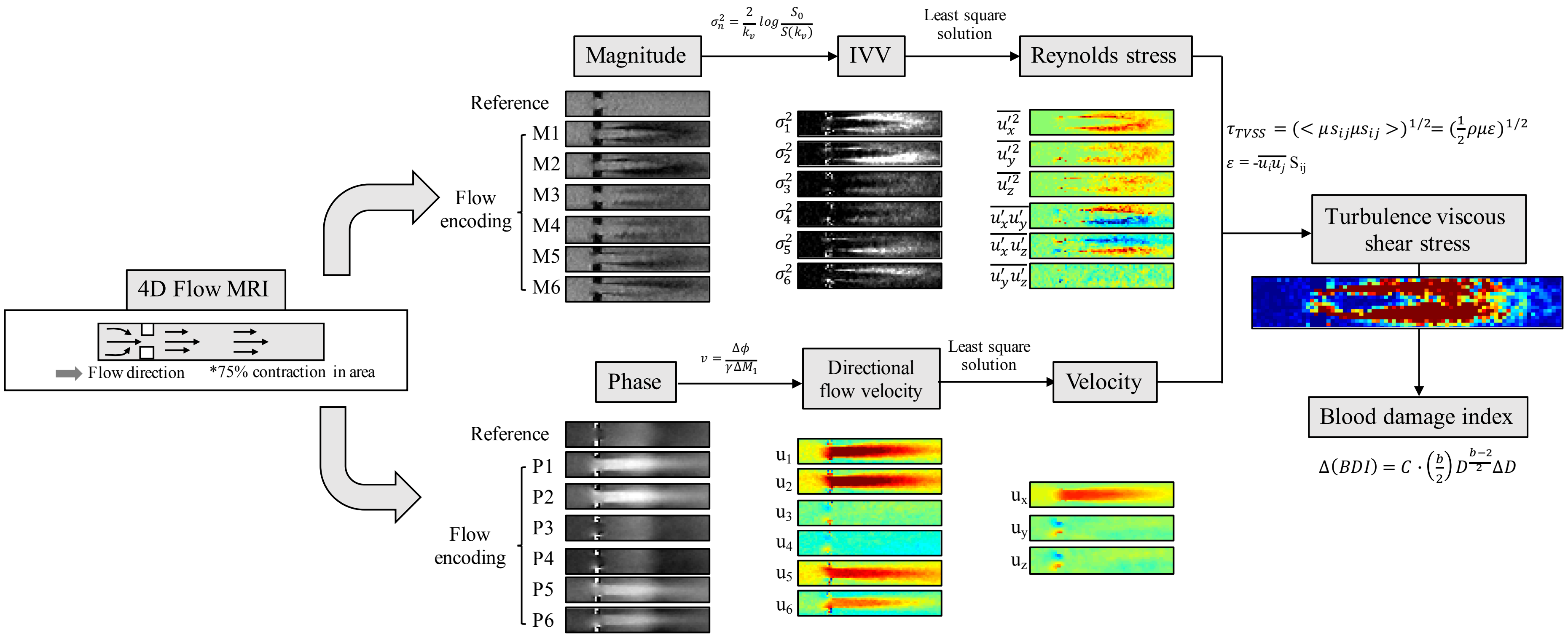

Figure S11. In-vitro demonstration of 4D Flow MRI with ICOSA encoding at Re ≈ 2000. The inset in 4D flow MRI shows the geometry of 75% constriction model for the experimental demonstration. IVV indicates the intravoxel variance, which is the square of the intravoxel standard deviation.

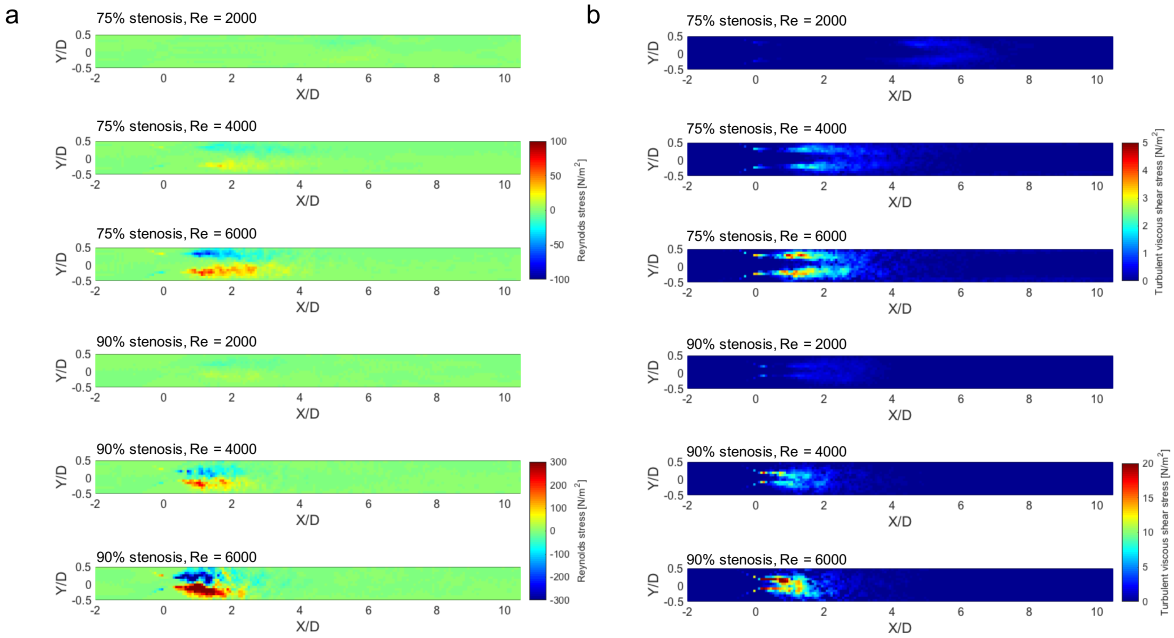

Figure S12. Comparison of Reynolds stress and TVSS at various Re and stensosis severisty (75% and 90% in area). (a) Reynolds stress component along XY direction (-$\rho\overline{u'v'}$). u' and v' are velocity fluctuation along X and Y axis, respectively. (b) TVSS distribution. X and Y are normalized by the upstream diameter (D = 14.6 mm). Principal flow direction is toward the positive X direction. The voxel size for MRI simulation was set to 1 mm. Note the color-maps for the 75% and 90% stenoses are different.

Supplementary Method

In-vitro demonstration of 4D Flow MRI with ICOSA encoding

Experimental measurement with 4D Flow MRI with ICOSA encoding was perform to confirm the feasibility of TVSS and BDI quantification. The stenotic phantom for the experiment was a sudden contraction/expansion model with 75% reduction. The upstream diameter without any constriction was 14.6 mm. The inlet and outlet upstream and downstream of the constriction was straight, which had the length of 1m to develop the Poiseuille flow upstream of the stenosis.

The working fluid was a blood analog composed of 26.4:73.6 glycerol/water mixture (by mass). The density and dynamic viscosity of the working fluid were 1056.2 kg/m^3 and 2.04×10^{-3} $kg \cdot m^{-1} \cdot s^{-1}$, respectively. The working fluid was circulated through the flow circuit system at a constant flow rate using a gear pump (ECO Gearchem G6, Pulsafeeder, NY). The flow rate was controlled from 1.31 to 7.61 L/min, which corresponds to Re of 989 to 5724. Re is expressed as Re = QD/(νA), where Q is the flow rate, D is the diameter of the channel, ν is the kinematic viscosity, and A is the cross-sectional area of the channel. The temperature of the fluid during the experiment was 22°C.

4D Flow MRI measurements were performed using a clinical 1.5T MRI scanner (1.5T Philips Achieva, Philips Medical Systems, Best, The Netherlands). A conventional gradient-echo sequence with asymmetric four-point flow encoding was modified to have six-directional icosahedral flow encoding, with one flow-compensated reference encoding. The velocity encoding parameter (VENC) was varied from 100 cm/s to 589 cm/s for the velocity measurements, and from 32 cm/s to 160 cm/s for the turbulence measurements, depending on the flow rate. Echo time and repetition time were 1.93–3.46 ms and 4.17–5.70 ms, respectively. The flip angle was 10°. The field of view was 336 mm × 336 mm × 180 mm with a 1 mm isotropic voxel size. Partial echo acquisition with a factor of 0.725 along the frequency-encoding directions was used, and the reconstructed with the zero-filling.

Three separate scans for each flow condition were measured. The first and second scans were performed with a different VENC to optimize the sensitivity of the velocity and the turbulence measurement, respectively. The third scan was performed with the same scan parameters as the velocity measurement, but with the pump turned off. The velocity field obtained from the third scan was used to correct the velocity offsets caused by background phase errors [1]. Estimation of three velocity components and Reynolds stress from 4D Flow MRI with ICOSA6 encoding is the same as described in the Method sections in main text,

Reference

1. Petersson, S. *et al.* Quantification of turbulence and velocity in stenotic flow using spiral three-dimensional phase-contrast MRI. *Magnetic resonance in medicine* **75**, 1249-1255 (2016).
